# Salt or fish (or salted fish)? The Bronze Age specialised sites along the Tyrrhenian coast of Central Italy: New insights from Caprolace settlement

**DOI:** 10.1371/journal.pone.0224435

**Published:** 2019-11-13

**Authors:** Luca Alessandri, Katia F. Achino, Peter A. J. Attema, Majoi de Novaes Nascimento, Maurizio Gatta, Mario F. Rolfo, Jan Sevink, Gianluca Sottili, Wouter van Gorp

**Affiliations:** 1 Groningen Institute of Archaeology, University of Groningen, Groningen, Netherlands; 2 Department of Prehistory, Autonomous University of Barcelona, Bellaterra, Spain; 3 Institute for Global Ecology, Florida Institute of Technology, Melbourne, Florida; 4 Department of Archaeology, University of York, York, England, United Kingdom; 5 Department of History, Culture and Society, University of Tor Vergata, Rome, Italy; 6 Institute for Biodiversity and Ecosystem Dynamics (IBED), University of Amsterdam, Amsterdam, Netherlands; 7 Earth Sciences Department, Sapienza University of Rome, Roma, Italy; University at Buffalo - The State University of New York, UNITED STATES

## Abstract

In 2017, an excavation led by the Groningen Institute of Archaeology and in collaboration with the Tor Vergata University of Rome, took place on two small islands in the Caprolace lagoon (Sabaudia, Italy), where Middle Bronze Age layers had previously been reported. Combining the results of an environmental reconstruction of the surroundings and a detailed study of the pottery assemblages, we were able to trace a specialised area on the southern island, in all probability devoted to salt production by means of the briquetage technique. The latter basically consists of boiling a brine through which a salt cake is obtained. The technique was widespread all over Europe, from Neolithic to Roman Times. Since the evidence points to an elite-driven workshop, this result has deep implications for the development of the Bronze Age socio-economic framework of Central Italy. Pottery evidence also suggests that in the Bronze Age sites along the Tyrrhenian coast of Central Italy where briquetage has already been hypothesised, more complex processes may have taken place. On the northern island, we collected a large number of so-called pedestals, which are characteristic features of briquetage, while chemical analyses point to salt or fish sauce production, like the roman *liquamen*, in a Middle Bronze Age domestic context.

## 1. Introduction

In 1991, Marco Pacciarelli first suggested that accumulations of reddish jar potsherds along the coast of northern Latium might evidence salt production by the briquetage technique [1]. Since then, many new sites have been put in relation with the exploitation of marine resources along the Central Tyrrhenian shores and (former) inland lagoons. However, some critical issues emerged in the interpretation of the evidence from these sites, which have not yet been resolved. In 2017, the Groningen Institute of Archaeology and the University of Rome Tor Vergata excavated a site on two small islands in the Caprolace lagoon. Accumulations of reddish jar potsherds were found and some little pedestals, which constitute a typical briquetage set [2]. The Caprolace evidence offers the opportunity to test the “salt hypothesis” by means of detailed ceramic study and archaeometric analyses and to shed new light on the interpretation of similar Tyrrhenian contexts.

## 2. Background and (proto)historical issues

### 2.1 Salt production in Europe

**Fig 1:**
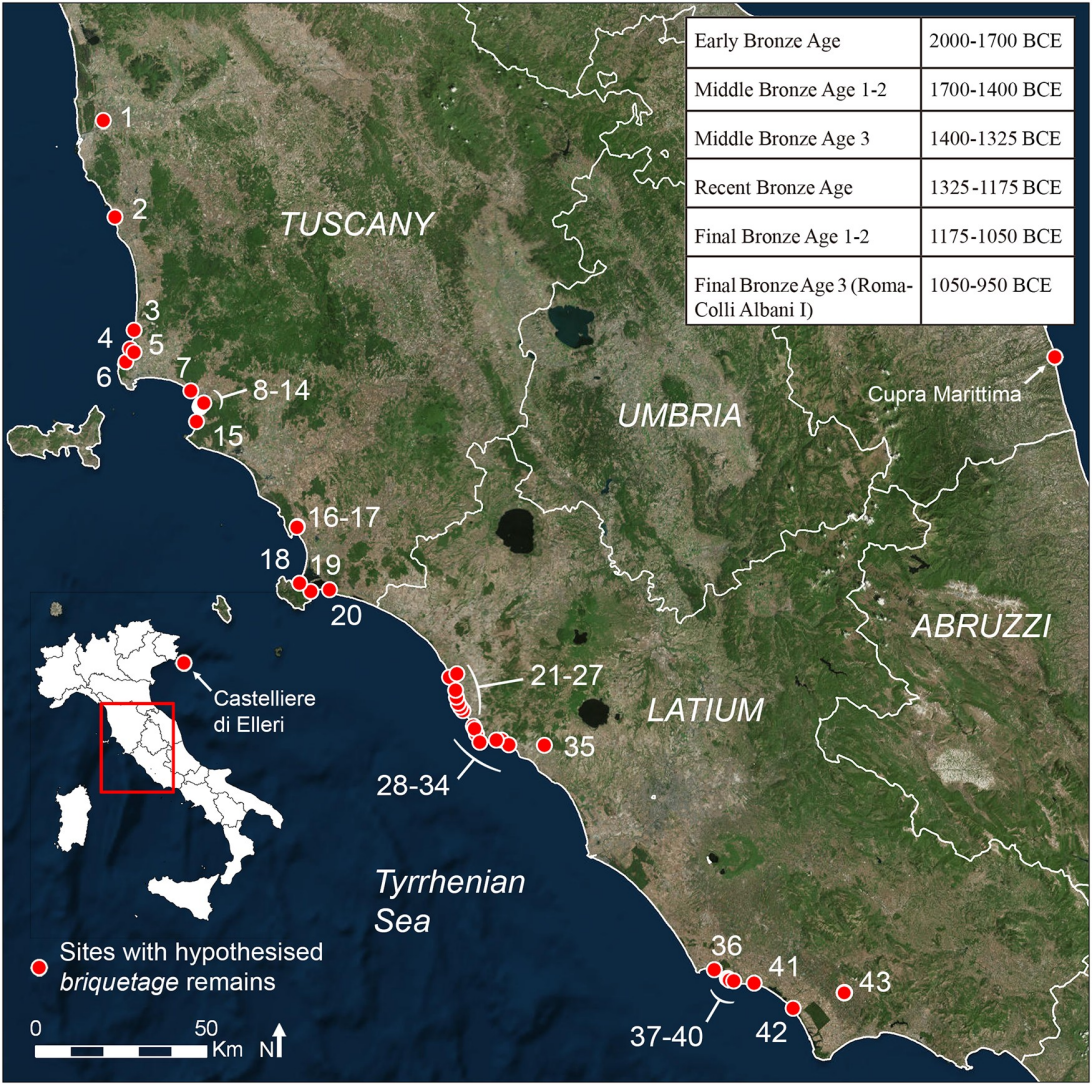
Sites along the Italian Tyrrhenian coast where briquetage salt-production has been hypothesised and a simplified chronological scheme for Central Italy Bronze Age. 1, Isola di Coltano; 2, Galafone; 3, Riva degli Etruschi; 4, Poggio del Molino; 5, La Torraccia; 6, Baratti; 7, Torre Mozza; 8, Puntone Nuovo-Le Chiarine; 9, Puntone Nuovo-Campo da Gioco; 10, Puntone Nuovo-Meleta; 11, Puntone Nuovo-Fiumara; 12, Puntone Nuovo-Fosso del Fico; 13, Portiglioni—Campo da Gioco; 14, Portiglioni; 15, Poggio Carpineta; 16, Tombolello; 17, Casa San Giuseppe; 18, Punta degli Stretti; 19, Poggio Pertuso; 20, Duna Feniglia; 21, Fontanile delle Serpi; 22, Le Saline di Tarquinia; 23, Bagni Sant’Agostino; 24, La Frasca; 25, Acque Fresche; 26, Torre Valdaliga; 27, La Mattonara; 28, Punta del Pecoraro; 29, Malpasso; 30, Marangone; 31, Torre Chiaruccia/Foce Guardiole; 32, Colonia dei Calabresi; 33, Quartaccia; 34, Grottini; 35 Greppa della Macchiozza; 36, Cretarossa/San Rocco (also known as Nettuno Depuratore); 37, Le Grottacce; 38, Pelliccione; 39, Saracca; 40, Area Stop 4; 41, Fosso Moscarello; 42, Caprolace; 43, La Cotarda. Refer to S1 Appendix for their chronologies and references. Aerial photo from Esri, Digital Globe, GeoEye, Earthstar Geographics, CNES/Airbus DS, USDA, USGS, AeroGRID, IGN, and the GIS User Community.

In 1991, the first protohistoric sites specialized in salt production were observed along the Tyrrhenian coast of Italy [1,3–5], near the modern town of Civitavecchia (Fig 1 and S1 Appendix, n. 21–31 and 33–34). The salt extraction process was reconstructed by comparison with examples of the so-called *atelier de briquetage*, found along the French Atlantic and Manche coasts [6–17] and in the Seille River Valley (Lorraine, France) [18–24]. In general, three relevant stages were identified in the process of salt-production: concentration, to obtain the brine, crystallization, to precipitate the salt, and conditioning, to mould the salt in cakes which can be stored and possibly transported and traded [25,26]. In the briquetage technique, the concentration stage probably consisted in letting the salt water naturally evaporate in artificial pits. The crystallization and the moulding stages comprised boiling of brine in specific vessels, which were then broken to extract the salt cakes. The archaeological evidence consists of kilns, pits, extended layers of charcoal, ash, and jar potsherds. Other features commonly associated with this production are the specific reddish colour of vessels, interpreted as a result of secondary firing, and the presence of pedestals and bars, used during the boiling process. The almost complete absence of vessel shapes typical of settlements, such as cups, bowls, and cooking stands, also constitutes indirect evidence for a specialized activity.

Evidence of briquetage is found throughout continental Europe [2,26,27], for instance in Germany [28–34], in Poland [35,36], in Spain [37–42], in Belgium and in the Netherlands [43–45].

The earliest occurrences of the *briquetage* practice are attested in the Chalcolithic sites of Cacica (Suceava) and Poiana Slatinei (Lunca, Moldavia), in Romania [46–49]. Additional sites occur in the Balkans, particularly the site of Provadia-Solnitsata (Bulgaria), where salt was produced using the boiling technique since the middle of VI millennium BCE [50–53]. The briquetage technique, for which the earliest evidence seems to come from eastern Europe, has its largest diffusion during the Iron and Roman ages, especially in northern Europe [2].

### 2.2 Salt production in Italy

Italian scholars adopted the typical assemblage of artefacts that was distinguished in earlier studies in Europe to identify *briquetage* activities, especially the presence of extensive pottery accumulations, which almost exclusively consist of reddish jar fragments [1,4]. Remains related to this production were first identified along coastal *Latium* (Fig 1, n. 21–31 and 33–34). Reddish jars and kilns used to boil brine were then identified at Baratti (Fig 1, n. 6), Puntone Nuovo (Fig 1, n. 8–12), Portiglioni (Fig 1, n. 13–14) and Duna Feniglia (Fig 1, n. 19–20). However, specific *briquetage* sets seem to be extremely rare. The typical pedestals, together with some characteristic half-conical vessels, were only observed along the Adriatic side of Italy, at the Castelliere di Elleri near Trieste [54,55] (Fig 1). Contrary, only few firebars were discovered at Coltano Island [56] (Fig 1, n. 1), Galafone [57] (Fig 1, n. 2) and Puntone Nuovo-Le Chiarine [58] (Fig 1, n. 8), in Tuscany. The votive deposit of Cupra Marittima in Marche region (Fig 1) deserves a special mention since here a symbolic representation of *briquetage* tools has been discovered among a large number of votive vessels dating to the archaic period (VI—early V century BCE) [59]. The characteristic half-conical vessels and pedestals are represented together with ceramic spoons, ladles and spatulas which, in the true dimensions, were possibly made of wood. If this interpretation is confirmed, the votive deposit of Cupa Marittima would shed new light on the *briquetage* process, as well as on potential connections with the sacred sphere. All the Italian sites are located along the coast and frequently are close to ancient lagoons. Most of the latter have been reclaimed during the early stages of the last century.

### 2.3 Critical issues regarding the “salt hypothesis”

The interpretation of these archaeological contexts as devoted to salt production is deficient in various respects [60]. The criticisms can be summarised as follows:

1. The reddish colour of vessels. Tencariu et al. [61] observed during an experiment, imitating the *briquetage* process, that jars undergoing a secondary firing under oxidative conditions turn red. Analyses of some potsherds from two Chalcolithic Romanian *briquetage* sites (i.e., Hălăbutoaia à Ţolici and Cacica) seemed to confirm the hypothesis that the red colour is due to such secondary firing [62]. Nevertheless, this hypothesis is contradicted by the discovery of a few tableware potsherds characterised by the same colour, which clearly have not been involved in such salt production process, in sites such as Pelliccione [63], Fosso Moscarello [64], and Saracca [64]. This evidence suggests that the reddish colour simply results from their primary production process, rather than to their successive use.
2. The vessels shape(s). Jars found in Italian *briquetage* contexts are often very different from those recovered in European sites. In Italy these are generally bigger, finely manufactured, with higher typological variability and often show thick cords, frequently notched, and big handles. Moreover, their shapes are very often inappropriate for an efficient evaporation process, although it stands to reason that some of them could have been used to other salt-related activities, like drying or storage.
3. The kilns. The kilns at Baratti Gulf and at Puntone Nuovo–Le Chiarine (kiln B) have been interpreted as salt production kilns [65]. The hypothesis is based on parallels from the *briquetage* ateliers of Enez-Vihan and Landrellec [13,15,16,66] on the northern coast of Brittany, dated to the II–I century BCE, and from the atelier of Moyenvic, Les Crôleurs, in the Seille valley [67] dated between Final Bronze Age (FBA) III and Ha C-D1. In particular, the use of stones and the parallel corridors seem to be quite common in salt kilns, especially in the area of the so-called *Briquetage de la Seille* (e.g the atelier Pransieu B, Ha C-D2/3), in Eastern France [19–21,68,69]. However, the presence of the cross-draft channel kiln is also attested in Greece, especially during the Neopalatial period, where they have been used for pottery production. These kilns constitute good parallels for the Baratti and Le Chiarine kilns, see for example the installations at Haghia Triada (Late Minoan IA) [70,71] and Kommos (Late Minoan IA) [72]. The structures found at Puntone Nuovo–Campo da Gioco and Portiglioni—Campo da Gioco have been interpreted as salt kilns too [5], but there are no good analogies at the moment.
4. Chemical analyses. At the moment, the preferred analytic method for assessing the use of the jars in the *briquetage* process relies on anomalous quantities/percentage of both chlorine (Cl) and/or sodium (Na) in the ceramic matrix. XRF (X-Ray Fluorescence) analyses revealed a high concentration of Cl in the pottery in which the brine was supposedly boiled at the sites of Champ-Durand (Vendée, France) and Barycz (Poland) [25,73–76]. Also, SEM-EDS (Scanning Electron Microscopy/Energy Dispersive X-ray Spectrometry) analyses revealed a relatively high concentration of both Cl (0.2–0.3 wt%) and Na (0.7–0.8 wt%), progressively decreasing from the interior surfaces towards the exterior, at the Chinese site of Zhongba [77]. A SEM-EDS analysis in a field experiment, aiming at reproducing the *briquetage* process, gave the same results, with Cl virtually absent at 2mm depth [61]. However, the only chemical analyses carried out so far in Italian *briquetage* sites are on 15 potsherds from Puntone Nuovo–Campo da Gioco. The XRDP (X-ray Diffraction Patterns) and SEM-EDS analyses failed to find salt (NaCl). In this case, the authors claimed that post-depositional processes, like leaching, dissolution or contamination, all related to meteoric or phreatic water, could have “washed out” the salt traces [5]. This post-depositional process, (i.e. the dissolution of salt crystals in water over time) has also been suggested by Horiuchi and colleagues [78] and Flad and colleagues [77].

### 2.4 Alternative hypothesis: Fish-processing

Several authors suggested an alternative interpretation for these archaeological sites, being fish-processing areas [79]. In particular, the site of La Mattonara [79–88], is interpreted as an area where jars and salt were produced, and fish was cooked [89]. Pacciarelli himself, who first suggested the “salt hypothesis”, does not exclude that at least part of the material could have been used for ancient fish processing [5]. In fact, since the fish spoils quite easily, some way of preservation was needed to allow its consumption at later stages and/or in distant markets. In general, ancient fish processing falls in two broad categories: dried, smoked, or salted fish (the latter: Greek τάριχος or Latin *salsamentum*) and various kinds of fish sauces (Greek γάρον or άλμη, Latin *garum* or *muria*, respectively, but also *liquamen* and *allec*) [90–92]. Unfortunately, some of these techniques, for example drying, are difficult to detect, since they don’t leave any physical evidence [93]. The oldest available written sources which describe the methods to obtain both salted fish and fish sauce are roman. The procedure to obtain *salsamentum* is partly explained by the Latin author Columella (I century CE) when he describes the method of salting pork as similar to those employed to salt fish (*Rust*. 12.55.4). It basically consisted of placing alternate layers of salt and previously gutted and cleaned portions of fish in a container, which is then topped up with a final layer of salt, pressed by weights. If the concentration of salt equals or exceeds 30% of the fish weight, the product can be kept inside the vessel for an indefinite time. As for the preparation of sauces, we know from several ancient authors that two generic methods existed: a slow and a quick one. A detailed recipe for preparing *liquamen* has been handed down by the Pseudo Gargilius Martialis (III century CE) in the *Medicina ex oleribus et pomis*. This procedure consists of packing alternating layers of herbs, fat fish and salt inside a well-pitched vessel. After seven days the content is stirred two or three times a day for another twenty days; afterwards, the liquid is collected and can be used to obtain *liquamen* or *oenogarum* adding wine, other herbs, spices, honey and boiling it in a bronze or iron vessel. The first part of the process takes usually place in the sun but, to speed up the process, artificial heating can be applied. The quick method is described in *Geoponica*, a X century CE collection of agronomy books, which may derive from a VI century CE Latin source [91,94]. A mixture of fish and oregano is placed in a vessel with brine and subsequently boiled. When the fish is cooked and the volume of the mixture is slightly reduced, the sauce is filtered until clear and then stored [95].

### 2.5 Evidence of fish-processing in the Mediterranean

In the Eastern Mediterranean, the earliest indirect evidence for fish-processing dates back to the Mesolithic (XI millennium BCE) [92,93,96,97]. Some evidence for the Bronze Age also comes from the Aegean region. In the cave site ‘tis Ouranias to Froudi’ (Crete), in 1962, a large quantity of Minoan salt (circa 1900, 1800–1600/1550 BCE) was recovered, which contained traces of organic residue. The salt was once put in the vessels in the form of layers, between which strata of organic carbonized compounds were visible [98]. Further evidence comes from the late Bronze Age settlement of Akrotiri (Santorini) [99] which was buried under the Thera eruption (1627–1600) [100,101]. In the settlement, a small storage vessel has been found, which contained a fish paste made from small fishes (picarels or bogues), small sting rays, cereal seeds and possibly other condiments. In the same settlement, several red porgy (*Pagrus pagrus*) bones inside a pithos have been interpreted as remains of a *allec*-like salted product. Finally, some articulated remains of common dentex (*Dentex dentex*) from the same location only had the first and the last vertebrae: they were probably opened along their belly, cleaned and subsequently salted or dried. However, in spite of these various examples, the Aegean archaeological fishbone assemblages are usually relatively small, do not show any evidence for processing and, from prehistory to Roman period, they are mostly composed (90%) of in-shore species [93]. Furthermore, no substantial fish-processing installations are known in the area [102,103]. In the western Mediterranean, industrial or quasi-industrial fish-processing for preservation is thought to have been introduced by the Phoenicians or the Greeks or both [91,104–106]. The first direct evidence comes from Punic layers in Cádiz, in Spain, where several sites began to operate in the V century BCE, till the end of the III century BCE [107,108]. However, in the Roman world saltfish trading already began in the VII century BCE but maritime and riverine cultures always employed different techniques for preserving fish on a small scale [90,109].

### 2.6 Evidence of fish consumption and processing in Italy

On the basis of the results from earlier excavations, it was generally assumed that in Italy fish consumption played a marginal role in the subsistence economy and diet during the Bronze Age and that fishing was essentially in-shore [110,111]. This is the case, for instance, in the Middle Bronze Age (MBA) settlement of Vivara Punta d’Alaca [112], where all the recognisable taxa were characteristic for coastal environments and no pelagic fish was present, and in other Calabrian [113,114], Apulian [115,116] and Marche [117] settlements. As for the exploited species, remains of tench (*Tinca tinca L*.), northern pike (*Esox lucius L*.), rudd (*Scardinius erythrophthalmus L*.), and chub (*Leuciscus cephalus L*.) have been collected in the Early Bronze Age (EBA) settlement of Canàr (Verona), near the Tartaro river. The same species, although in different percentages, have been found in the FBA layers of the nearby settlement of Frattesina (Rovigo), which is located along the banks of a Po paleochannel [118,119]. In the settlement of Stagno di Livorno (FBA, Livorno), which was at the border of an ancient brackish lagoon [120], half of the identifiable fish remains belonged to mullet (*Mugil sp*.). Also, seabream (*Diplodus sp*.), gilthead seabream (*Sparus aurata L*.), European eel (*Anguilla anguilla L*.), European seabass (*Dicentrarchus labrax L*.), smooth-hounds (*Mustelus sp*.) and pandora (*Pagellus sp*.) were present. Most of them are euryhaline species, which can tolerate different salt concentrations, which would confirm that most of the catch came from the lagoon [110]. Fishing in a fluvio/lagoon system is also attested in the M. Ingino settlement in Central Italy (Perugia). Here some remains of eels and cyprinids were collected [121]. As for sea fishing, few species have been recovered from southern Italian sites: two vertebrae of a probable smooth-hounds and some remains of a sea turtle in the MBA layers in Monopoli (Bari); gilt-head bream and groupers from the MBA settlement of Punta le Terrare (Brindisi). Mullet, grouper and cuttlefish were exploited in the settlement of Roca Vecchia (Lecce) [115]. In the settlement of Vivara Punta d’Alaca, stingray (*Dasyatis sp*.), seabream (*Diplodus sp*.), sand steenbras (*Lithognathus mormyrus L*.), salema (*Sarpa salpa L*.), gilthead seabream (*Sparus aurata L*.), porgy (*Sparidae indet*.), porgy/picarel (*Sparidae/Centracanthidae indet*.), scorpionfish (*Scorpaena sp*.), stargazer (*Uranoscopus scaber L*.), wrasses (*Labridae indet*.), grouper (*Serranidae indet*.) and bony fish (*Osteichthyes indet*.) were present [112]. Another line of evidence might come from the stable isotopes (δ^13^C, δ^15^N and δ^34^S) although very few analyses have been done so far on Italian Bronze Age communities [122,123]. Most of them show a predominantly terrestrial diet with the only exception of the EBA (circa 2100–1750) Grotta dello Scoglietto burial site [124,125], which shows a possible contribution of fresh-water fish [126]. However, evidence of fish processing has never been found in Bronze Age Italian sites.

## 3. Material and methods

### 3.1 Description of the site

**Fig 2:**
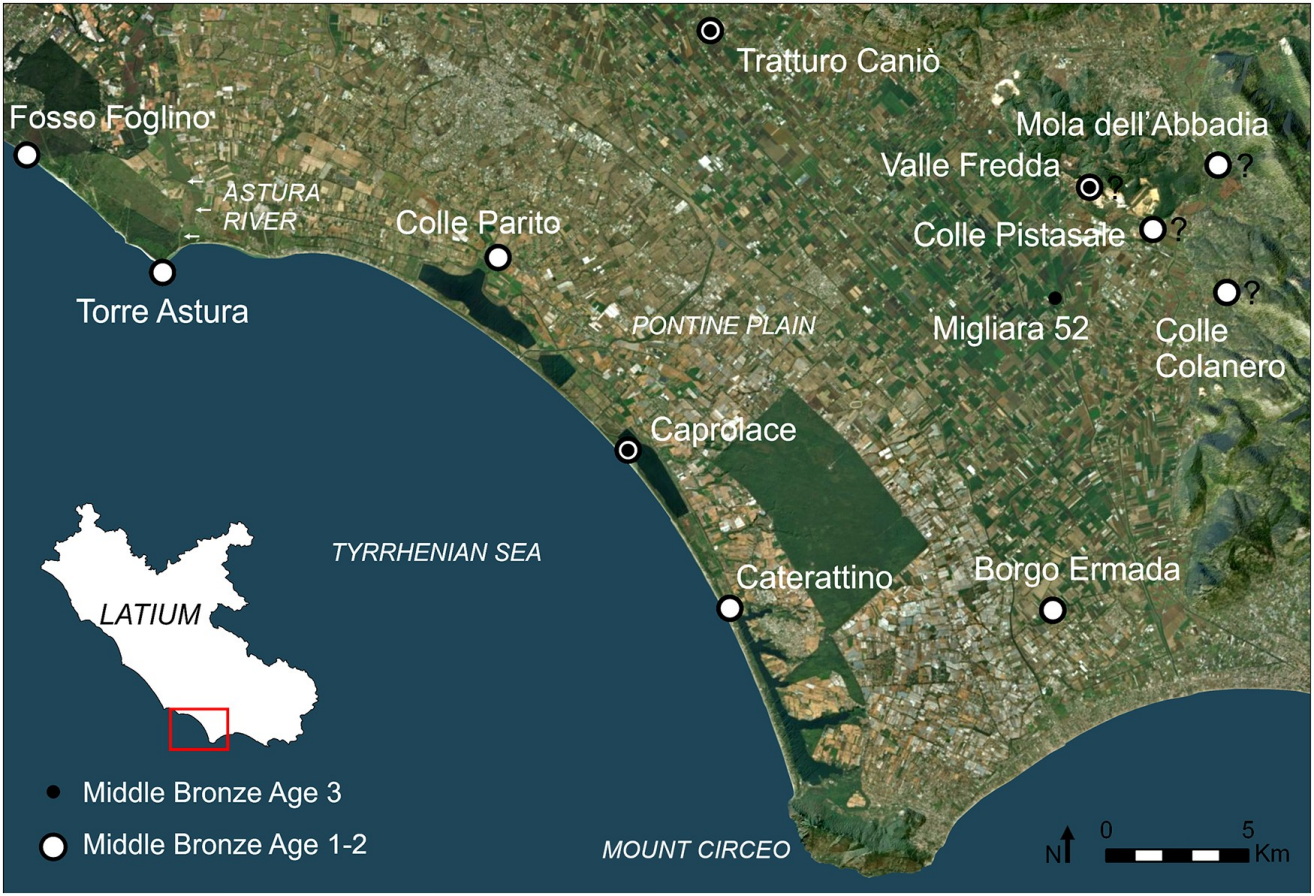
The MBA sites in the Pontine Plain. Aerial photo from Esri, Digital Globe, GeoEye, Earthstar Geographics, CNES/Airbus DS, USDA, USGS, AeroGRID, IGN, and the GIS User Community.

All necessary permits were obtained for the described study, which complied with all relevant regulations. Field work and archaeometric analyses were conducted under permits issued by the Ministero dei Beni e delle attività culturali e del turismo, Direzione generale archeologia, belle arti e paesaggio (permits number: DG-ABAP 9557 and 6155-P). The individual pictured in S7 Appendix has provided written informed consent (as outlined in PLOS consent form) to publish their image alongside the manuscript. The Caprolace lagoon is located in the National Park of Circeo (Sabaudia), between the Mount Circeo and the mouth of the River Astura (Fig 2; coordinates UTM WGS 84, 33T: E329611, N4580258). Nowadays, the lake has an open connection with the sea and is saline. It has an artificial rectangular shape extending for around 2,3 kmq (226 hectares) and reaches a maximum depth of 3m. The shape and original depth of the lake have been strongly altered during the land reclamation carried out in the Pontine Plain between 1930 and 1940 [127]. In the northern part of the Caprolace lagoon, two small islands occur. Abundant Bronze Age and Roman pottery was recovered in the water along the eastern shore of the northern (larger) island by locals and during an archaeological survey carried out in 2007 [64]. During the latter, potsherds were also recovered, though in lesser quantities, along the whole perimeter of the southern (smaller) island.

### 3.2 Excavation campaign

**Fig 3:**
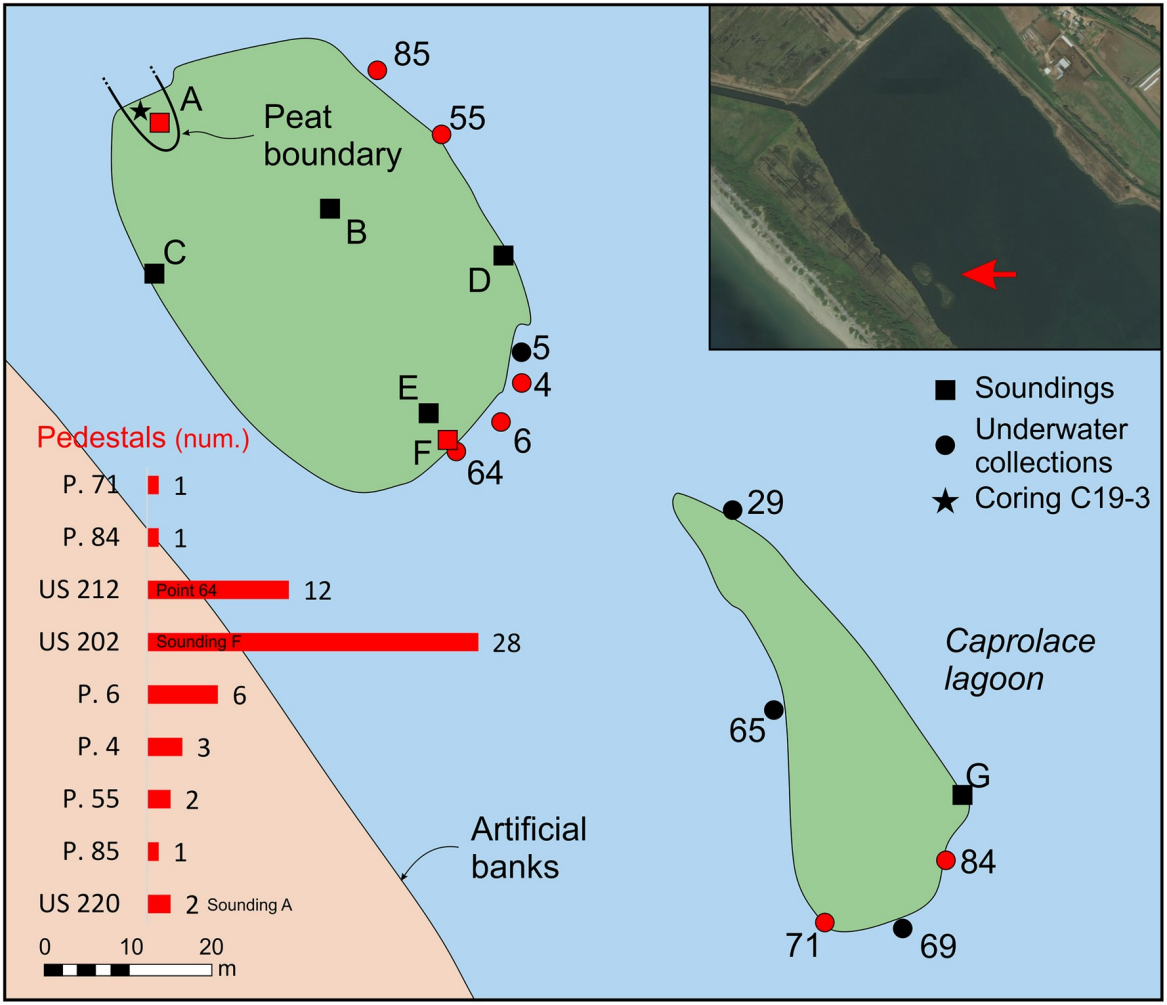
The stratigraphic soundings, the underwater collections and the corings, with the number of recovered pedestals. Drawing: L. Alessandri; upper right image from Esri, Digital Globe, GeoEye, Earthstar Geographics, CNES/Airbus DS, USDA, USGS, AeroGRID, IGN, and the GIS User Community.

In summer 2017, an archaeological campaign was undertaken to fully establish the archaeology of the islands and its context. The lagoon is a Site of Community Importance (n. IT6040012), so we had to respect the rules imposed by the authorities to protect the reserve. Therefore, six soundings were carried out along the perimeter and on the northern island, and one on the southern island, while artefacts were collected in the adjacent shallow shore waters at different spatial areas (Fig 3). The soundings, measuring about 2,5 m^2^, were studied for their stratigraphy and the finds discovered were recorded on a grid system. Soil samples of each layer were collected. In this paper, only the relevant contexts will be discussed, namely the Bronze Age deposits from soundings F and G and the Stratigraphic Unit (SU) 212. All the collected archaeological finds are stored at the Archaeology Laboratory of the University of Tor Vergata (Rome, Italy).

### 3.3 Environmental reconstruction

Systematic corings have been performed along transects on both small islands to establish their sedimentary built-up. The dominant sediment type encountered was well-sorted medium to coarse sands without visible stratification. The depth of the current groundwater level was found to be directly linked to the level of the lagoon and was visible as a transition from greyish-yellow sand to truly grey, reduced sand. The corings reached least 1.3 m depth and, wherever sediments other than well-sorted sands were encountered, were extended into the underlying sands. Coring was by Edelman corer, and by gauge auger for organic sediments and below the groundwater level.

In the NW part of the northern island, corings showed the presence of a small elongated depression, filled with peat and thinly covered by younger sands. Cores were taken for ^14^C dating and study of the paleoenvironmental conditions. From core C19–3, small sediment sub-samples were taken by cutting the core in 2 cm thick slices. In the field, a change in composition had been observed at about 92 cm depth, with a transition from peat to grey slightly sandy peaty clay. To further study this change in composition, sub-samples were briefly boiled with 5% KOH and sieved over a 100 μm sieve. After handpicking plant macrofossils from these fractions for ^14^C dating, they were dried and weighed. Potentially datable plant macro remains were encountered in samples from between 98 and 70 cm depth and identified under the microscope. From these, two samples (76–78 cm and 94–96 cm) were selected for ^14^C dating. For samples from the same depths, slides were prepared by embedding suspended sediment in a Naprax medium, for microscopic study of diatoms and other microfossils present. Lastly, a thin section was produced for study of the sediment characteristics in the core between 85 and 92 cm, by embedding the sediment in resin, followed by cutting and polishing to a thickness of ca. 30 um.

### 3.4 Archaeometry: Analytical methods and sample preparation

Elemental mapping at the microstructural level and microtextural features, including estimates of the percentage abundance and dimensions of individual components in pottery, were obtained from thin section electron-optical images that were collected by a FEI Quanta 400 and a Leo1455VP scanning electron microscopes (SEM) equipped with energy dispersive X-ray spectrometry (EDS). Microprobe analyses on glasses and mineral phases were performed at the CNR-Istituto di Geologia Ambientale e Geoingegneria (Rome, Italy) by a Cameca SX-50 EMP, equipped with five wavelength-dispersive spectrometers, using 15 kV accelerating voltage, 15 nA beam current, 10 μm beam diameter and 20 s counting time. Operating conditions were set to 15 kV accelerating voltage; 15 nA beam current; 10–15 μm beam diameter; 20 s per element counting time; Wollastonite (Si and Ca), corundum (Al), diopside (Mg), andradite (Fe), rutile (Ti), orthoclase (K), jadeite (Na), phlogopite (F), potassium chloride (Cl), barite (S), and metals (Mn) were used as standards. The Ti content was corrected for the overlap of Ti-Kα peaks. In order to evaluate the accuracy of the electron microprobe analyses, three international secondary standards (Kakanui augite and rhyolite RLS132 glasses from the United States Geological Survey) were measured prior to each analytic run.

For SEM-EDS analyses, ~5 mm-thick slices of pottery were cut perpendicularly to the surface of vessels, thus obtaining analyzable areas covering approximately 1 cm × 5 cm areas. For EMPA (Electron microprobe analysis) analyses, thin-sectioned slices of pottery were dry-cut normally to the surface of vessels, consolidated in epoxy resin and polished with progressively finer pastes on one side to obtain a thickness of about 0.03 mm and analyzable areas of ~4 cm2. All polished surfaces were then coated with carbon in a carbon-coater to ensure conductivity.

## 4. Results

### 4.1 Environmental reconstruction

Corings were systematically carried out across both islands (S9 Appendix), but apart from a small elongated depression filled with peat in the northwestern part of the northern island, the subsoil everywhere consisted of well sorted dune to beach ridge sand (S10 Appendix). In the depression the maximum thickness of the peat was ca.100 cm. Its location and extension are indicated in Fig 3, which also shows the location of the archaeological test pit A, in this part of the island. Observations in this pit show that the natural sedimentary sequence has been completely disturbed up to a considerable depth. In fact, only Roman Republican layers with Black Gloss potsherds, amphorae and tiles fragments were present until a depth of 95 cm, although with some residual Bronze Age potsherds. The excavation ended at this depth due to the infiltrations of water. The core studied in detail (C19–3) is just outside the disturbed area, as could be established by later coring, which showed that the core was at least about 25 cm outside the Roman disturbance.

The transition between rather pure peat and more clayey sediment was observed at about 30 cm below the current ground water level in all corings that were performed in the peat filled depression, suggesting that it is linked to a major change in environmental conditions. However, from the sediment analyses and thin section it appeared that the presumed clayey texture of the upper part of this core–above ca. 90 cm depth–was due to the abundant presence of diatoms and not from silt or finer sized detrital material, which is virtually absent. In other words, the observed transition reflects a transition towards truly diatomitic sediment, though the amount of sand size material also increases.

Hand-picked plant macro remains that could be identified were encountered in the subsamples between 92 and 98 cm and were mostly seeds from *Cladium mariscus* and *Ruppia maritime*, of which the latter is truly indicative for salt marshes [128]. This is very much in line with the results from the diatom analyses (S6 Appendix), which for both samples show a dominance of brackish to marine taxa (e.g. *Cyclotella quillensis*). Moreover, the diatom analyses point to a higher water level for the upper sample, with more planktonic taxa, which suggests that the rather sharp transition in sediment composition is probably due to an increase in water depth.

Results from the ^14^C analyses are 1606–1433 cal BCE for the sample from 94–96 cm depth (a piece of wood) and 1303–1407 cal AD for the sample from 76–78 cm (charcoal fragments) (S5 Appendix). The first date fits very well in the MBA lifetime of the site. In fact, according to the few available radiocarbon dates, the MBA in central Italy should start around 1750 BCE with an ending at around 1400 BCE [129]. On the other hand, the second sample is surprisingly young. The observed ^14^C age of the charcoal from 76–78 cm might very well be explained by rather recent bioturbation, instead of late medieval disturbance of the topsoil. Such significant bioturbation, presumably by earthworms, has recently been described for the nearby Astura valley, where small Early Modern charcoal fragments were found at depths of several metres in clearly earlier sediments [130].

The observations and analyses thus point to the presence of a small depression with a salt marsh with initially rather shallow brackish to truly saline conditions and a transition to a deeper lagoon with still saline conditions around 1500 cal BCE. In other words, most probably a lagoon which was open to the sea and with a water level that probably rose to a higher level later on. This rather excludes the existence of a well-developed coastal dune system as is present today, in which the lagoon was completely closed before the nineteenth century’s coastal hydraulic works.

### 4.2 Pottery typo-chronology and contexts

**Fig 4:**
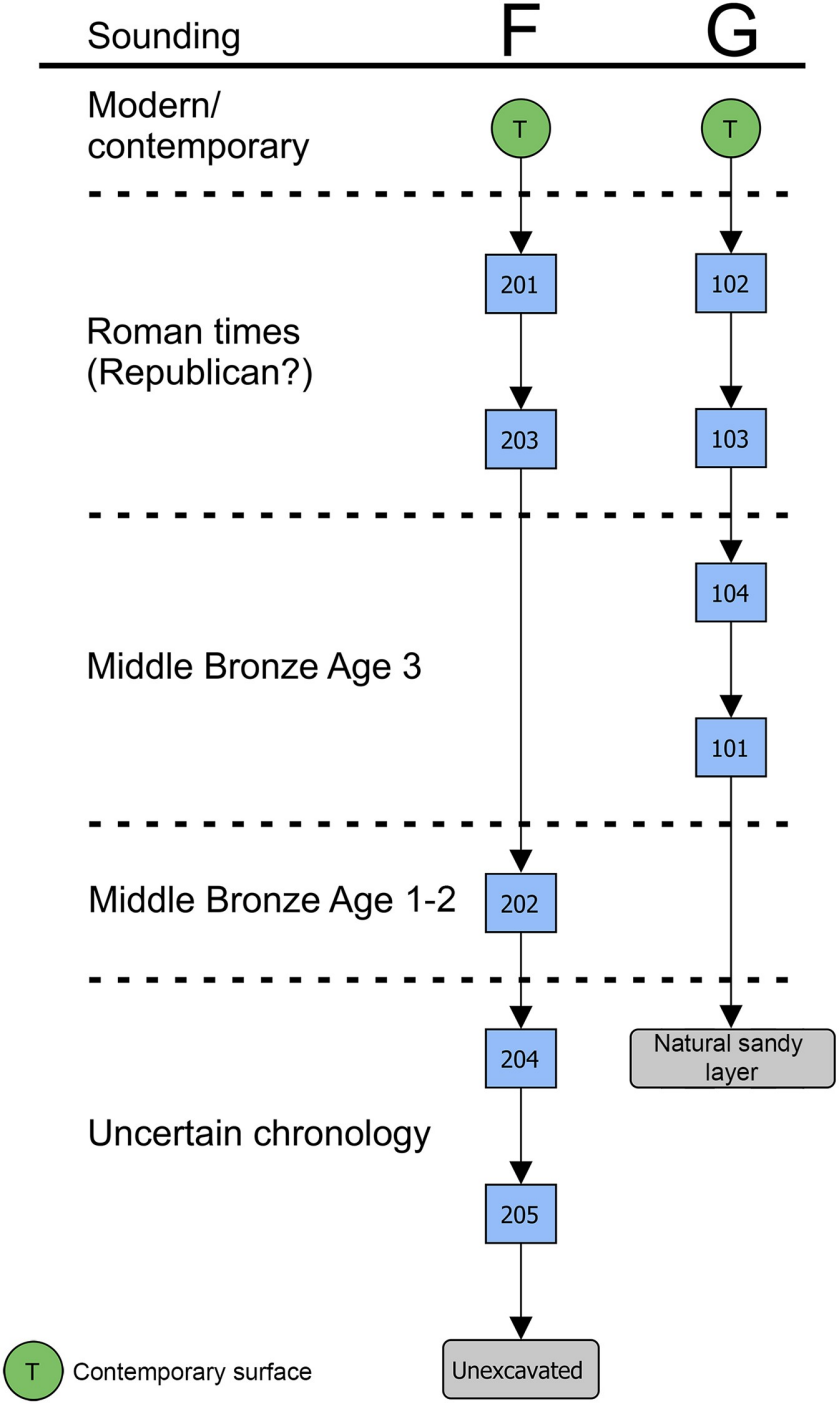
Matrix of soundings F and G.

**Fig 5:**
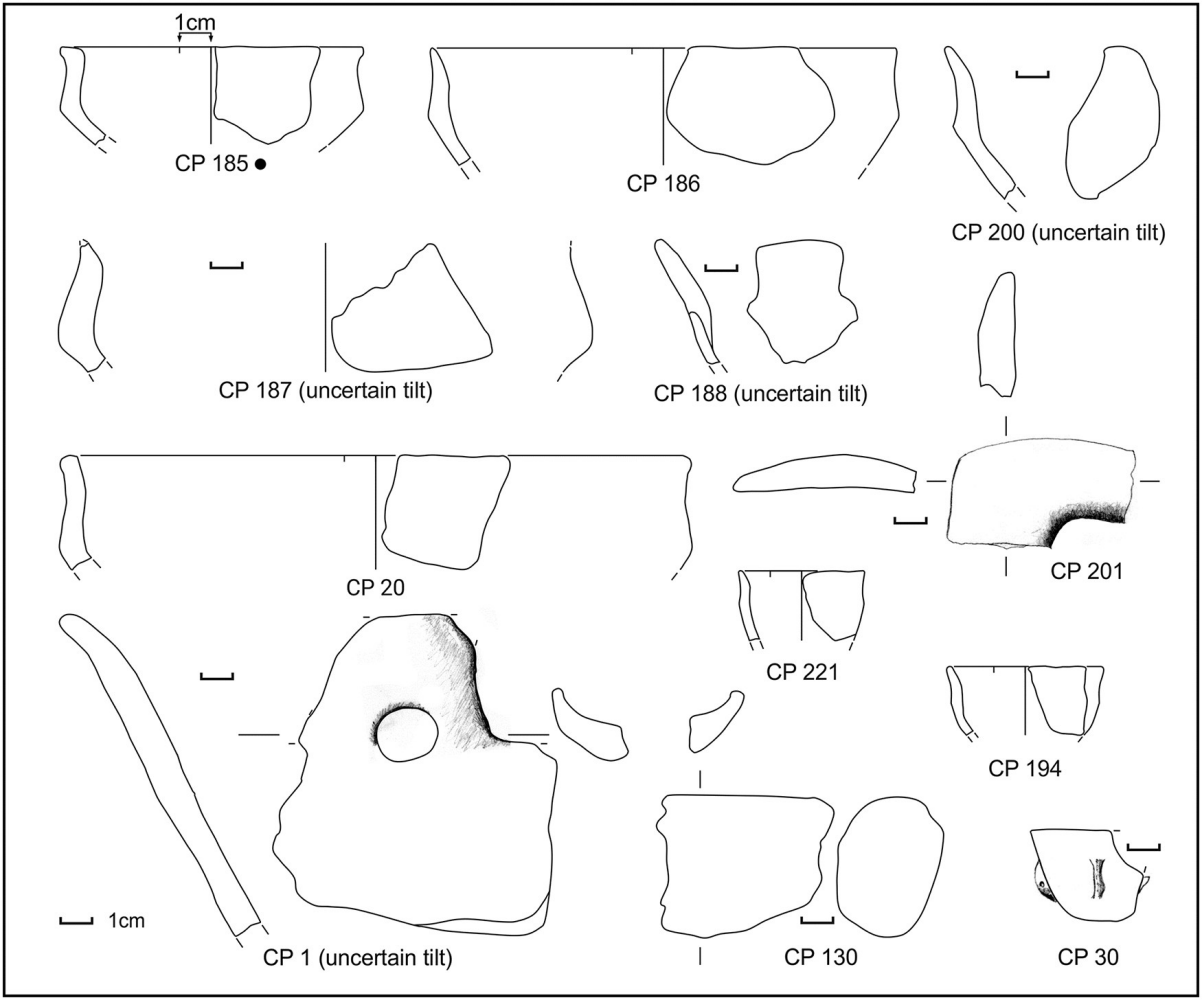
Potsherds from SU 202, open shapes. Black dots: sampled for physicochemical analyses. Drawings: L. Alessandri.

**Fig 6:**
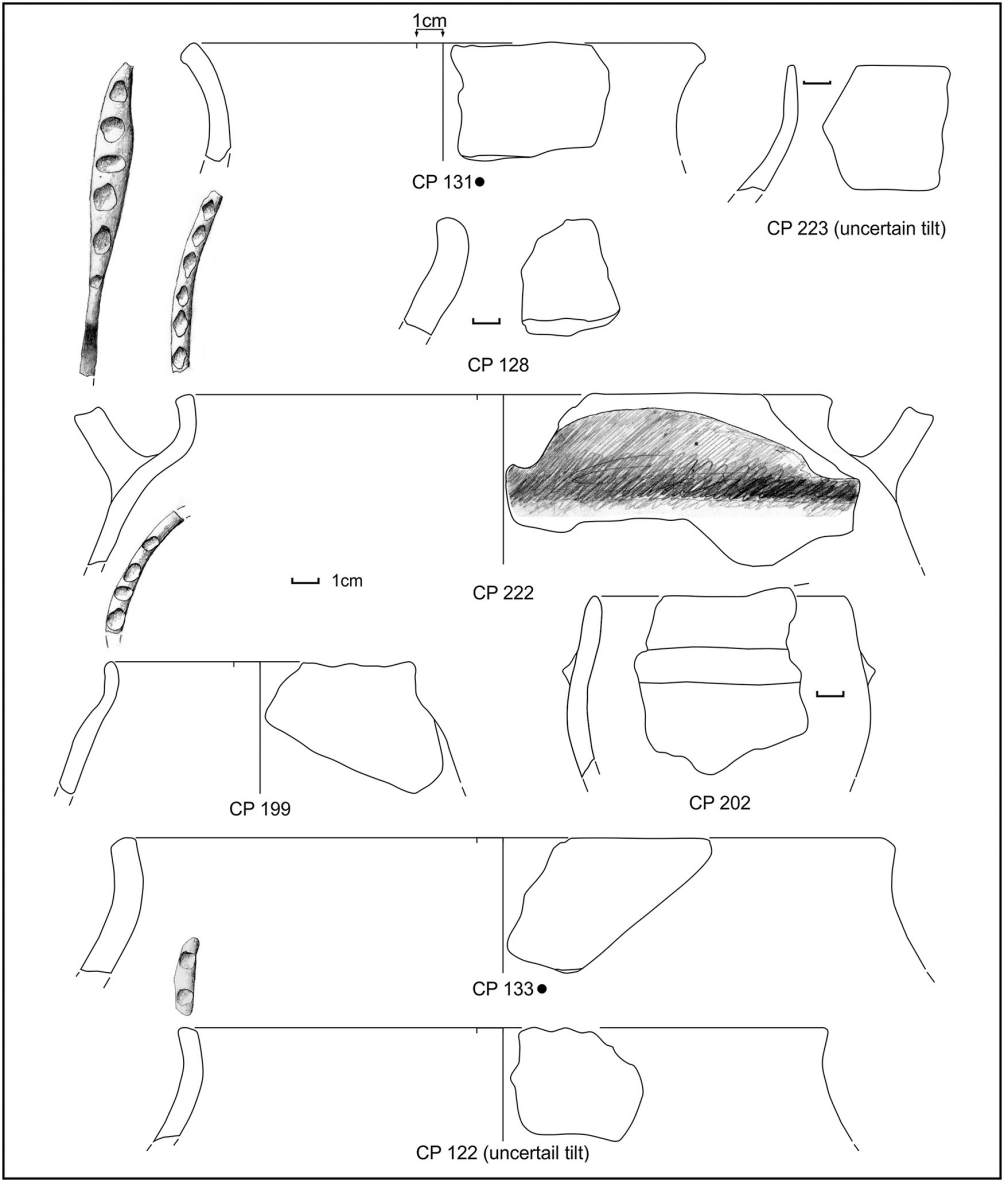
Potsherds from SU 202, closed shapes. Black dots: sampled for physicochemical analyses. Drawings: L. Alessandri.

**Fig 7:**
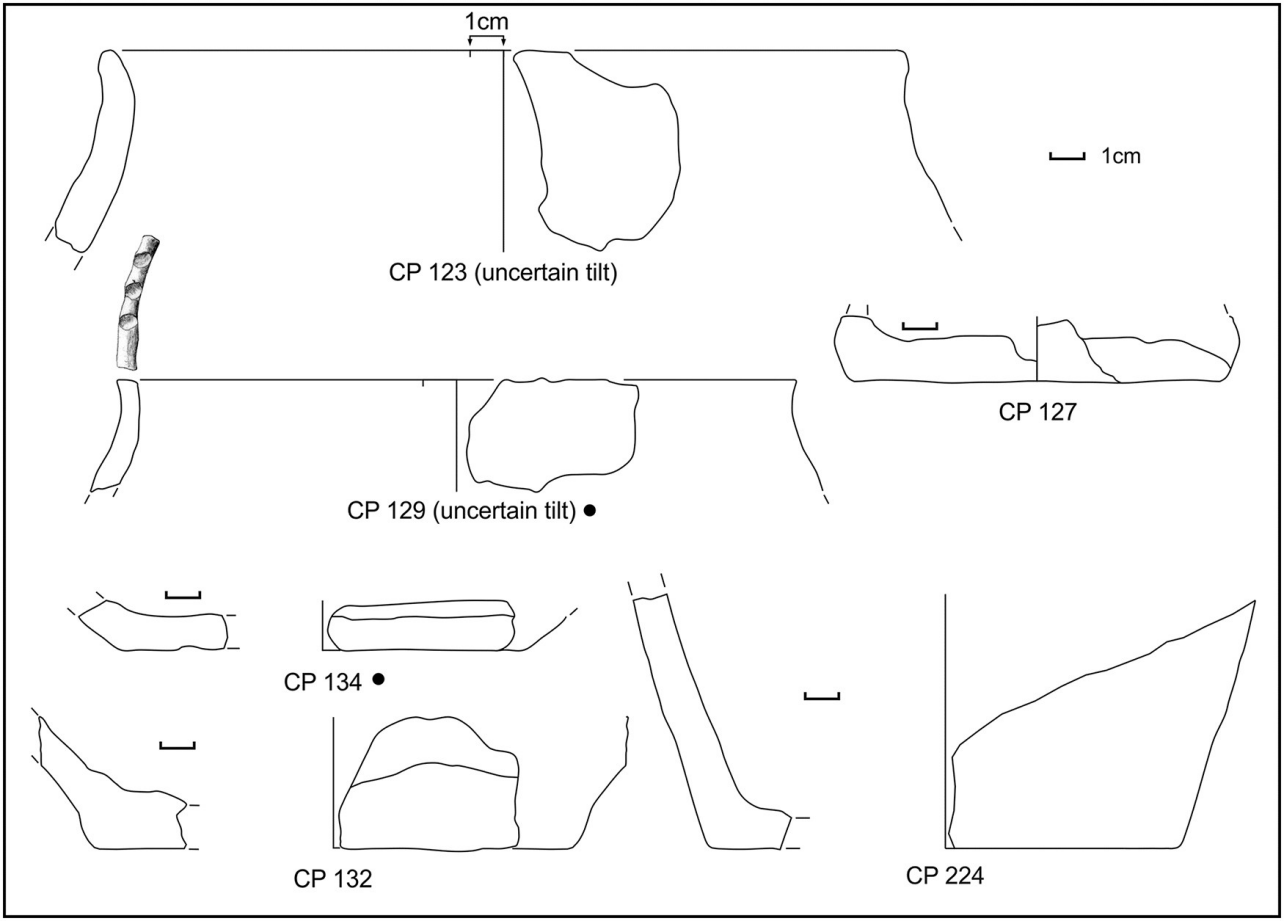
Potsherds from SU 202, closed shapes and bases. Black dots: sampled for physicochemical analyses. Drawings: L. Alessandri.

In sounding F (Fig 3), beneath the top layers (SUs 201 and 203) containing very few Roman potsherds (Fig 4), abundant MBA impasto potsherds, sub-phases 1 and 2A, have been found in SU 202 (Figs 5–7 and S7 and S8 Appendices).

**Fig 8:**
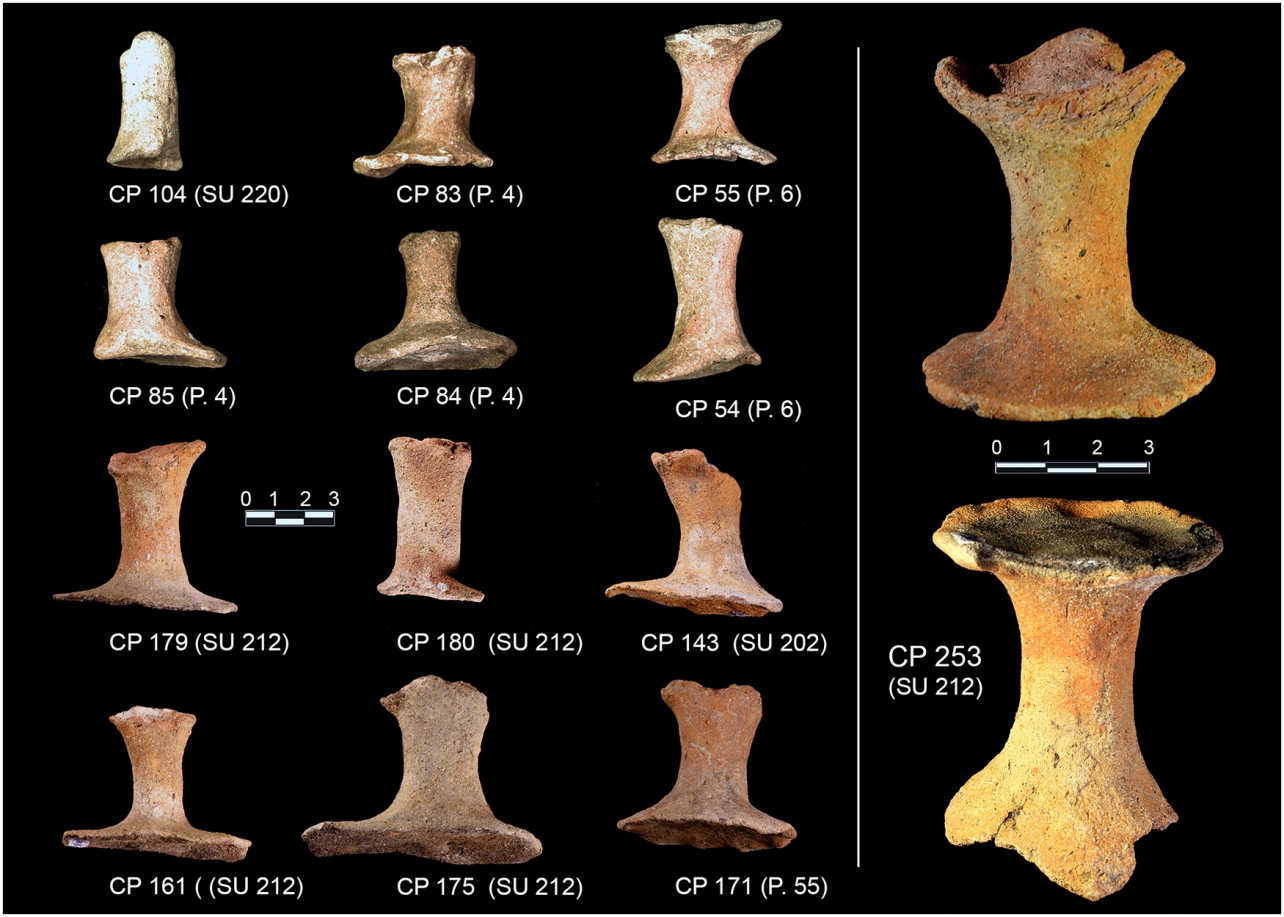
A selection of the pedestals found on the northern island. Photo by A. Ferracci and M. F. Rolfo.

Good parallels can be found in several Italian Bronze Age settlements (S2 and S3 Appendices) belonging both to the MBA Grotta Nuova (approximately Emilia Romagna and central Italy) and Protoappenninico (southern Italy, with the exception of Calabria) cultures [3,129,131–133]. Within this layer, several pedestals, which are usually found in European salt-production sites (*briquetage*), have also been collected (Fig 8). Parallels can be found, for example, in the France sites of La Pâture à Vache, La Bruyère [8] and Barriéra cave (La Turbie) [134], where they are called ‘piliers’, in the German settlement of Brehna [135] and also in the English site of Brean Down [136]. The pedestals from Caprolace have a slightly rounded base with a concave centre and, where (rarely) preserved, a set of three very rudimentary lobes on the top. The shank is circular in section. Such features are usually interpreted as pedestals on which the vessels were placed during the brine evaporation or drying/moulding process [8,137,138].

The potsherd assemblage of SU 202 points to a domestic context, due to the presence of a considerable amount of bowls and cups (used for drinking and eating) (CP 185–188, CP 200–202, CP 1, CP 20), although large jars (CP 122–123, CP 128–129, CP 133, CP 222 and probably all the bases) presumably for grain or water storage and small jars (CP 199, CP 131) are also present. In SU 202, the percentage of bowls and cups in the total ceramic assemblage is 50% (9 vessels, not counting the bases). For a comparison, in the EBA hut F from Filobraccio Filicudi [139], and in the MBA “sub-rectangular hut” in Coppa Nevigata, Manfredonia [140], bowls and cups form 49% (116 vessels) and 65% (27 vessels) of the total. Vice-versa, in a specialised context like the FBA Pelliccione, bowls and cups form 19% (28 vessels) [63]. However, it must be noted that SU 202 was only partially excavated and its true extent is unknown. Thus, it is not possible to know whether our sample is statistically representative. CP 130 might be interpreted as a cooking stand bar and it has good parallels with sub-type 1F of the Moffa’s typology for the southern Italy cooking stands [141]. Type 1 is widespread from the MBA until the Iron Age. Alternatively, it can be interpreted as a salt stove bar, similar to those collected, for example, in the two ateliers Pransieu A and Digue in the French municipality of Marsal [21,142]. The miniaturised vessels (CP 30, CP 194, CP 221) are usually interpreted in the Italian literature as ritual/votive objects. Indeed, they have been found in great number in nondomestic contexts, like caves and lakes [143,144]. However, they have also been collected inside domestic contexts like the EBA Delta IV hut of the Lipari acropolis settlement [145] and the EBA Hut F of the Filo Braccio settlement in the Filicudi island [139], where they might have been used as children’s toys [146]. Very few potsherds, undiagnostic, have also been found in the layers below (SUs 204 and 205).

**Fig 9:**
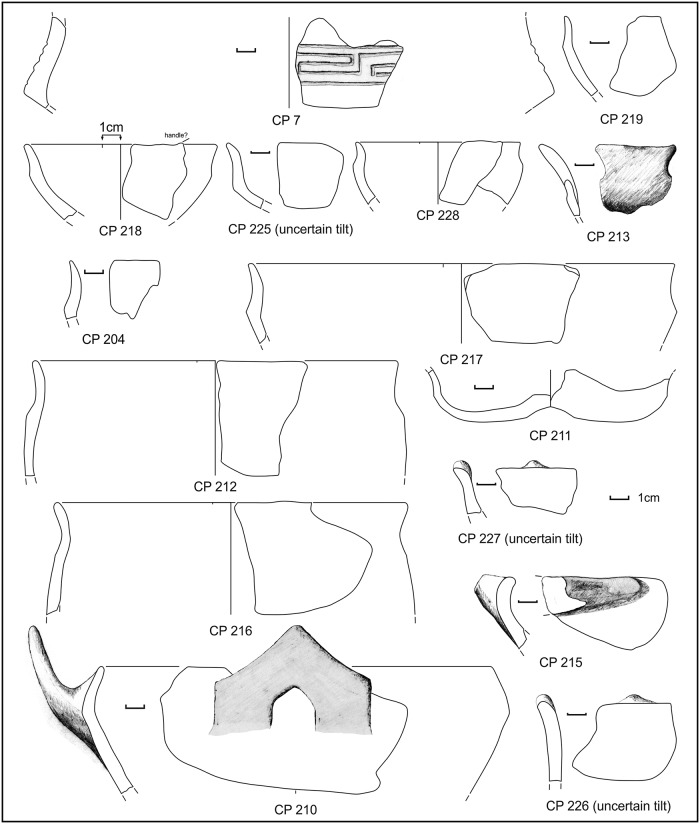
Potsherds from SU 212, open shapes and base. Drawings: L. Alessandri.

**Fig 10:**
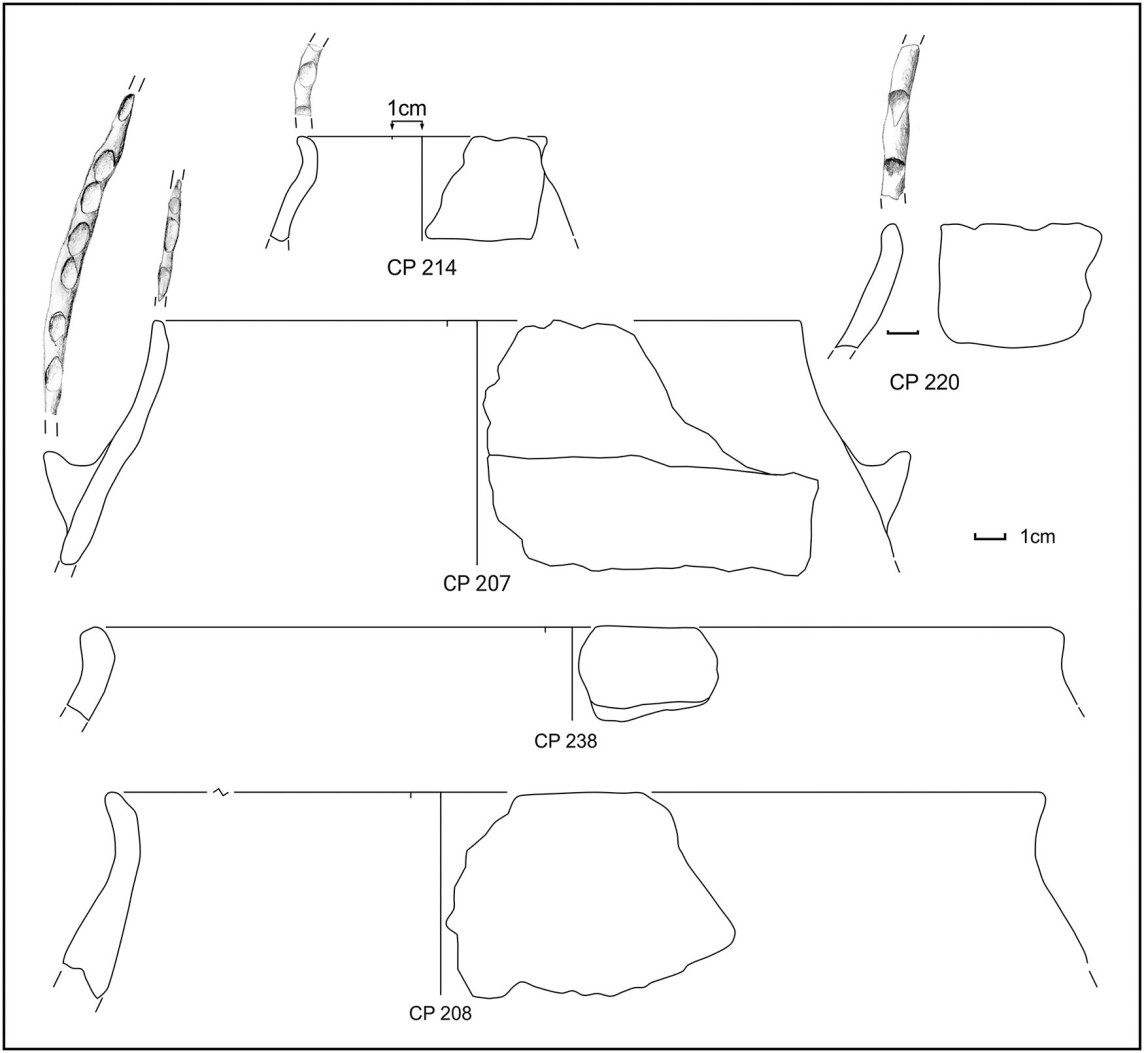
Potsherds from SU 212, closed shapes. Drawings: L. Alessandri.

During manual corings around the northern island, we detected a layer full of similar potsherds (point 64 or SU 212) in the water, just south of sounding F, near the already exposed profile. Further manual corings were made to check for the composition and depth of this layer, which was clearly visible in the shallow water and turned out to be almost exclusively made of potsherds (80% on total) and to be ca. 20cm thick. We were able to detect this layer in a zone of about 1m, all around the northern island (Fig 3, points 4–6, 55, 85), with the exception of the western side where the extremely muddy lake bottom prevented any exploration in the water. The collected impasto potsherds, mostly from point 64 (SU 212), were strongly eroded and can be dated to MBA subphases 1 and 2, with very few MBA 3 fragments (Figs 9 and 10 and S3 Appendix). Several pedestals were also found (Fig 3).

**Fig 11:**
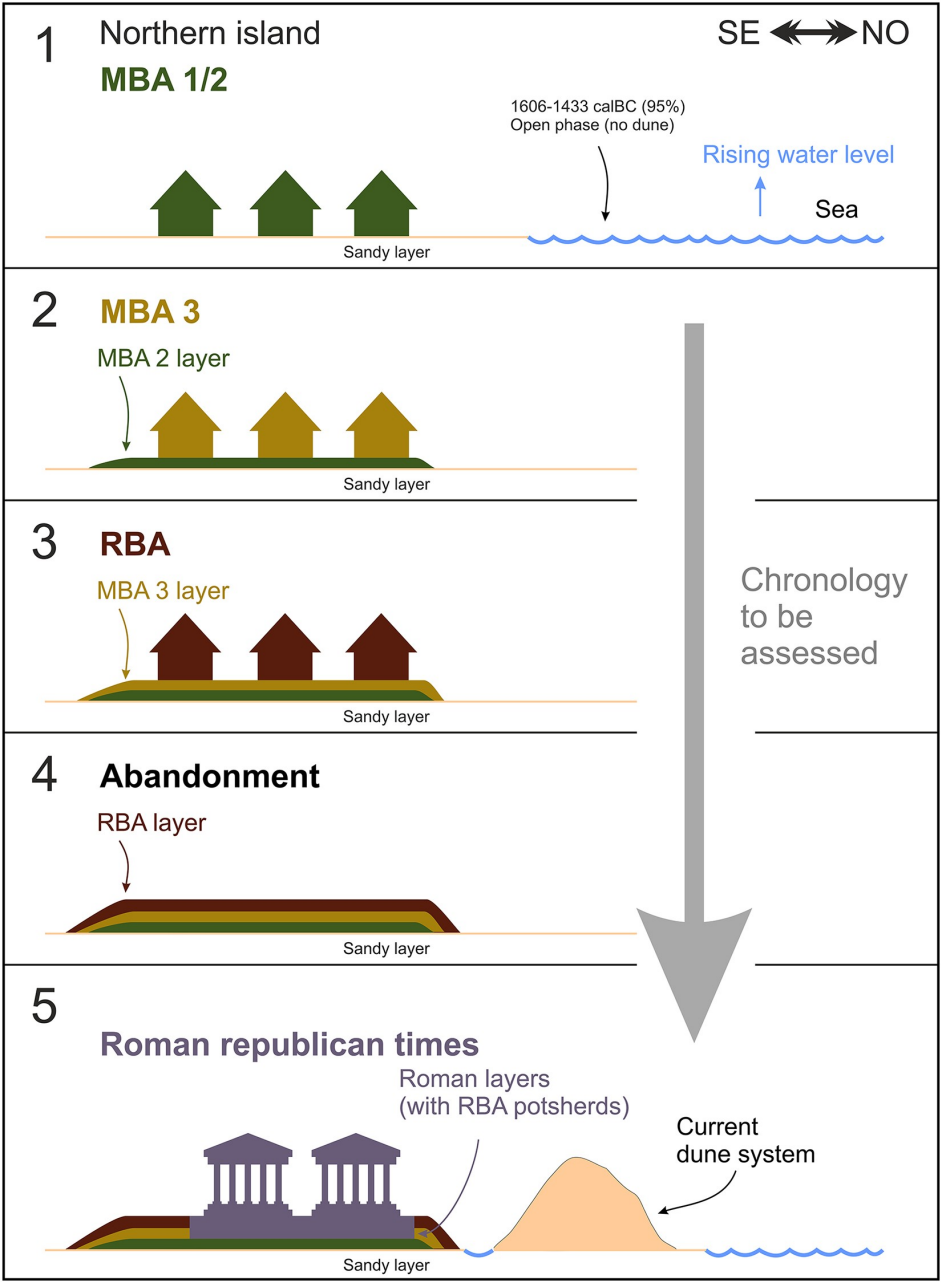
The reconstructed development of the northern island from Bronze Age to Roman times.

**Fig 12:**
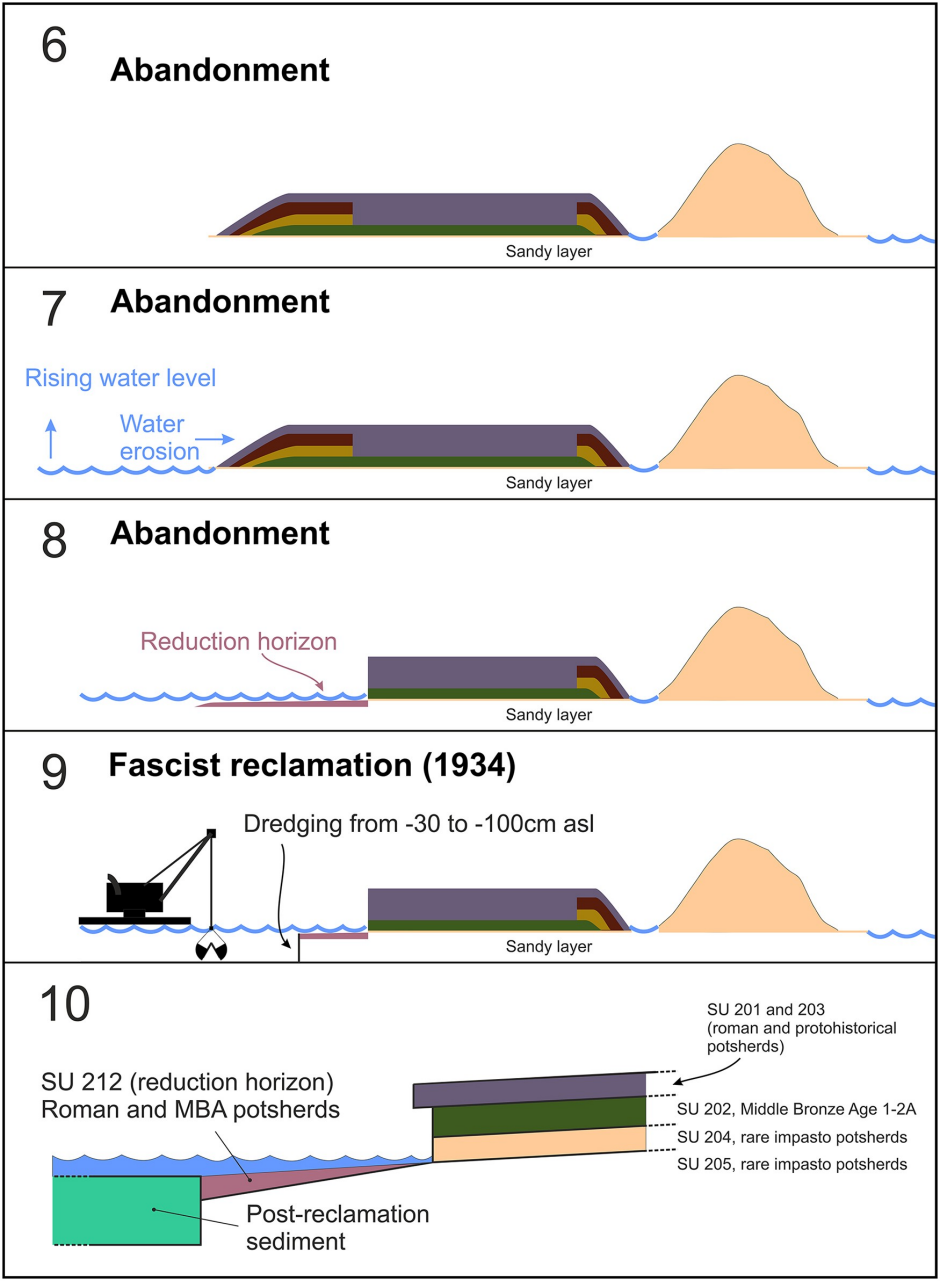
The reconstructed development of the northern island from Roman times to date.

We interpret the layer as a “reduction horizon”: the result of the erosion and the contemporary removal of the finer (sand) fractions of the sediment by wave action, along the foreshore (Figs 11 and 12).

In these layers, the objects made of organic matter are not usually preserved; only the harder material can survive, like ceramic and stones. Similar layers have been observed in the Bronze Age settlements around the lake Neuchâtel [147] and the Lake Geneva [148]. So, basically, SU 212 might be an eroded and reworked SU 202 with very few Roman potsherds, which come from the layers above SU 202: SU 201 and SU 203. The ceramic assemblage of the layer (see also the potsherds published in [149], which have been partly collected in the same place) is very similar to that of the SU 202. The percentage of bowls and cups in the total ceramic assemblage is 70%, thus pointing at a domestic context. The presence of the layer a few centimetres below the lake bottom also implies that the foreshore, and thus the lake level, was lower at the time of the layer formation. This would also confirm the already discussed reconstruction inferred from the coring in the northern island. Nonetheless, a lower lake level would be consistent with the reconstruction of the relative sea-level changes [150–152]. A recent research focused on the reconstruction of the Pontine Plain paleogeography places the relative sea-level at the end of the EBA at -1.5m [153]. Finally, the layer was truncated by the dredging of the lake in 1934, at which time the islands were recorded in the reclamation maps, so it is now only preserved in a “doughnut-shape” about 1m wide, around the northern island.

**Fig 13:**
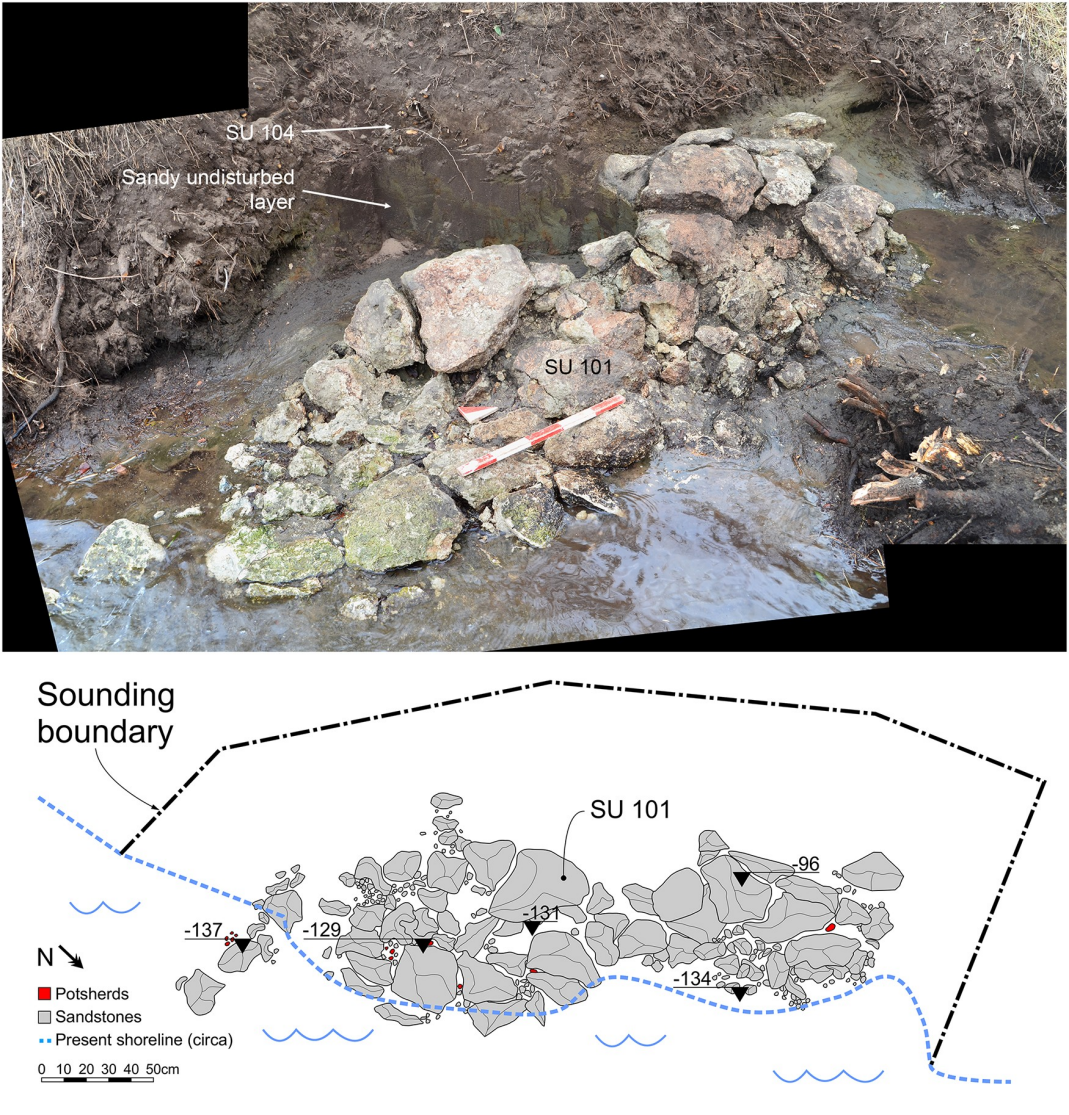
The dry stone wall in sounding G (southern island). Picture obtained by merging two different images using Microsoft Image Composite Editor. Drawing: G. Presti, A. Ferracci, D. Santinelli.

In the small southern island, the remains of a short dry stone wall composed of very porous sandstones (SU 101, Fig 13) have been discovered during the excavation of sounding G. The structure continues in the water and below the bottom of the lake, which would confirm that the lake level was lower than today.

**Fig 14:**
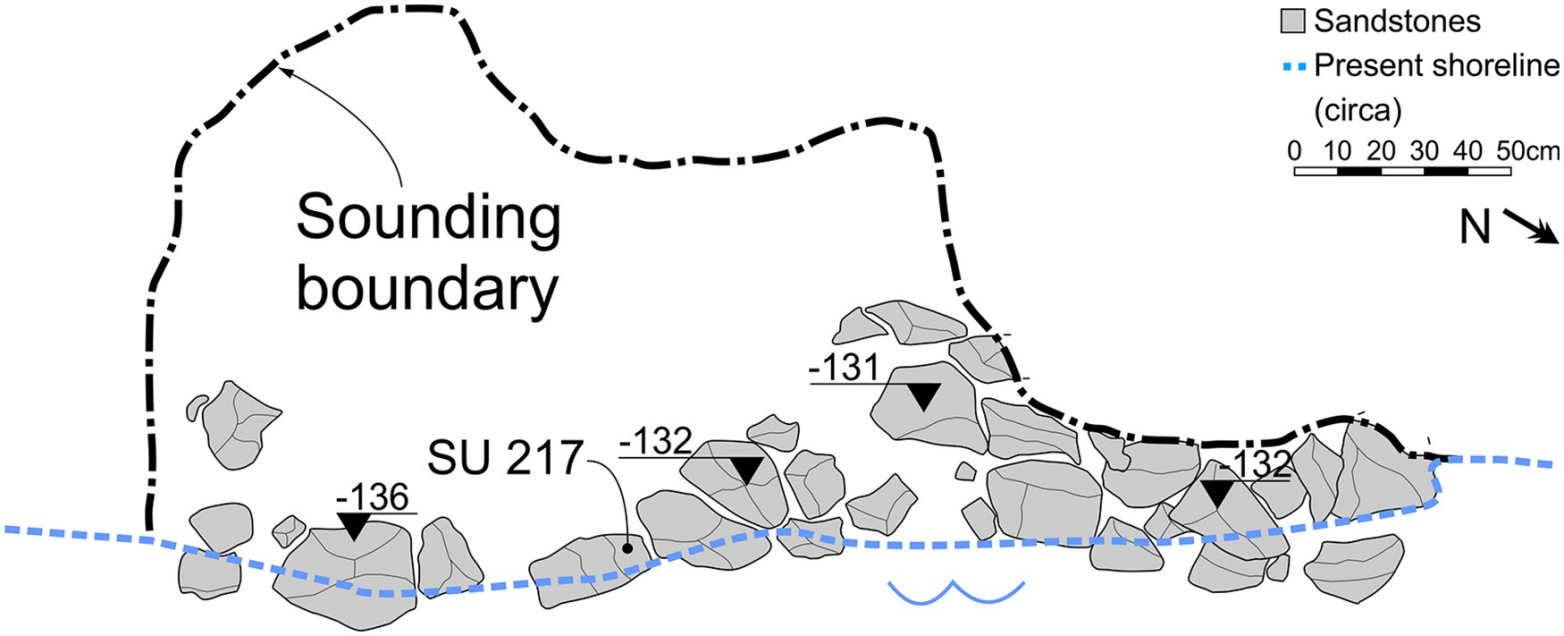
The dry stone wall in sounding D (SU 217, northern island). Drawing: L. Tomassi, G. Presti, M. De Marzi.

Other dry stone walls made of the same sandstone have been found in two other spots around the larger island (Fig 3, sounding D and point 55) and they were placed near the current shoreline, partly in the water. We were not able to further investigate the feature in point 55, but we could excavate the structure in sounding D (Fig 14, SU 217).

**Fig 15:**
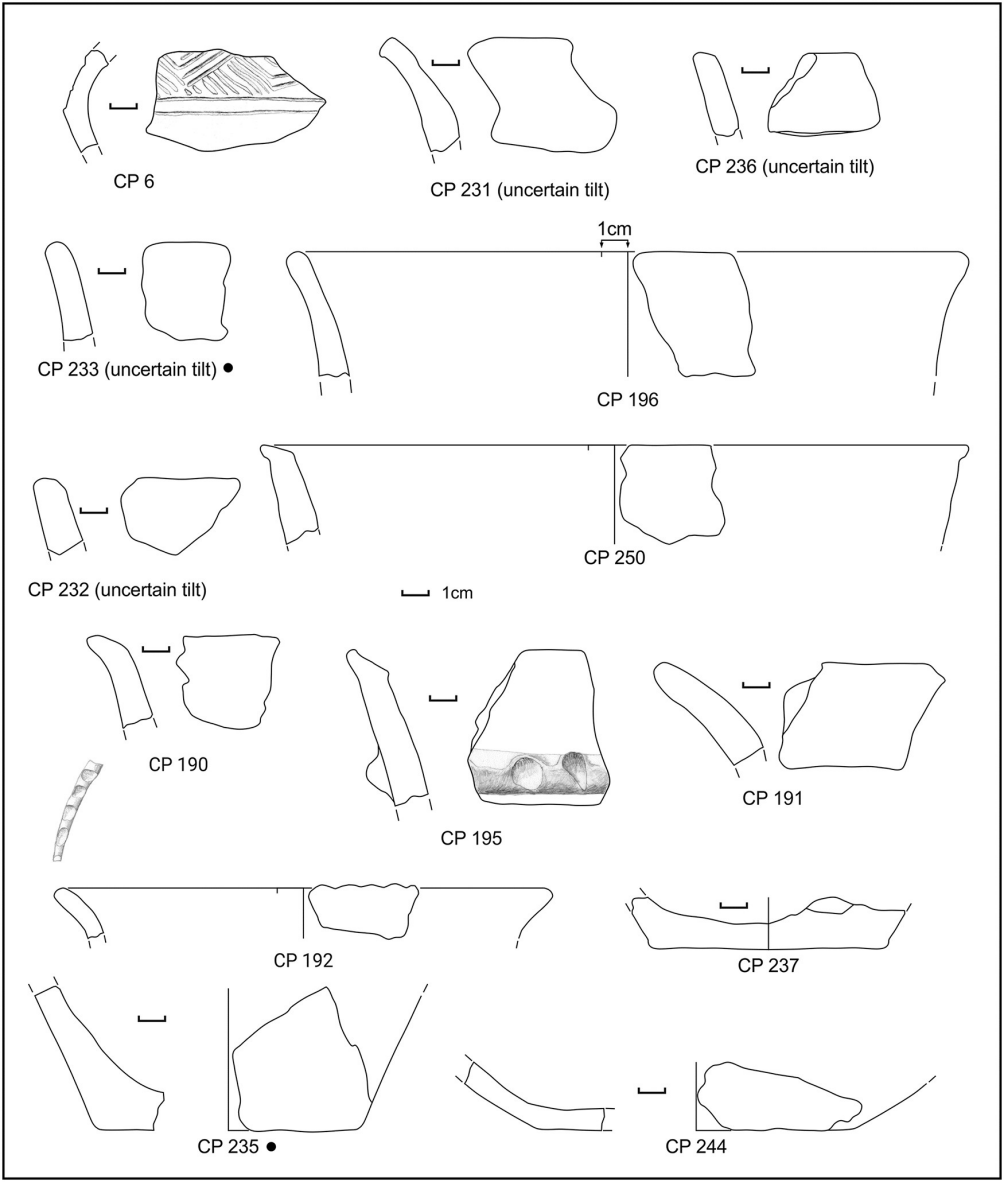
Potsherds from SU 104, the decorated sherd, closed shapes and bases. Drawings: L. Alessandri.

**Fig 16:**
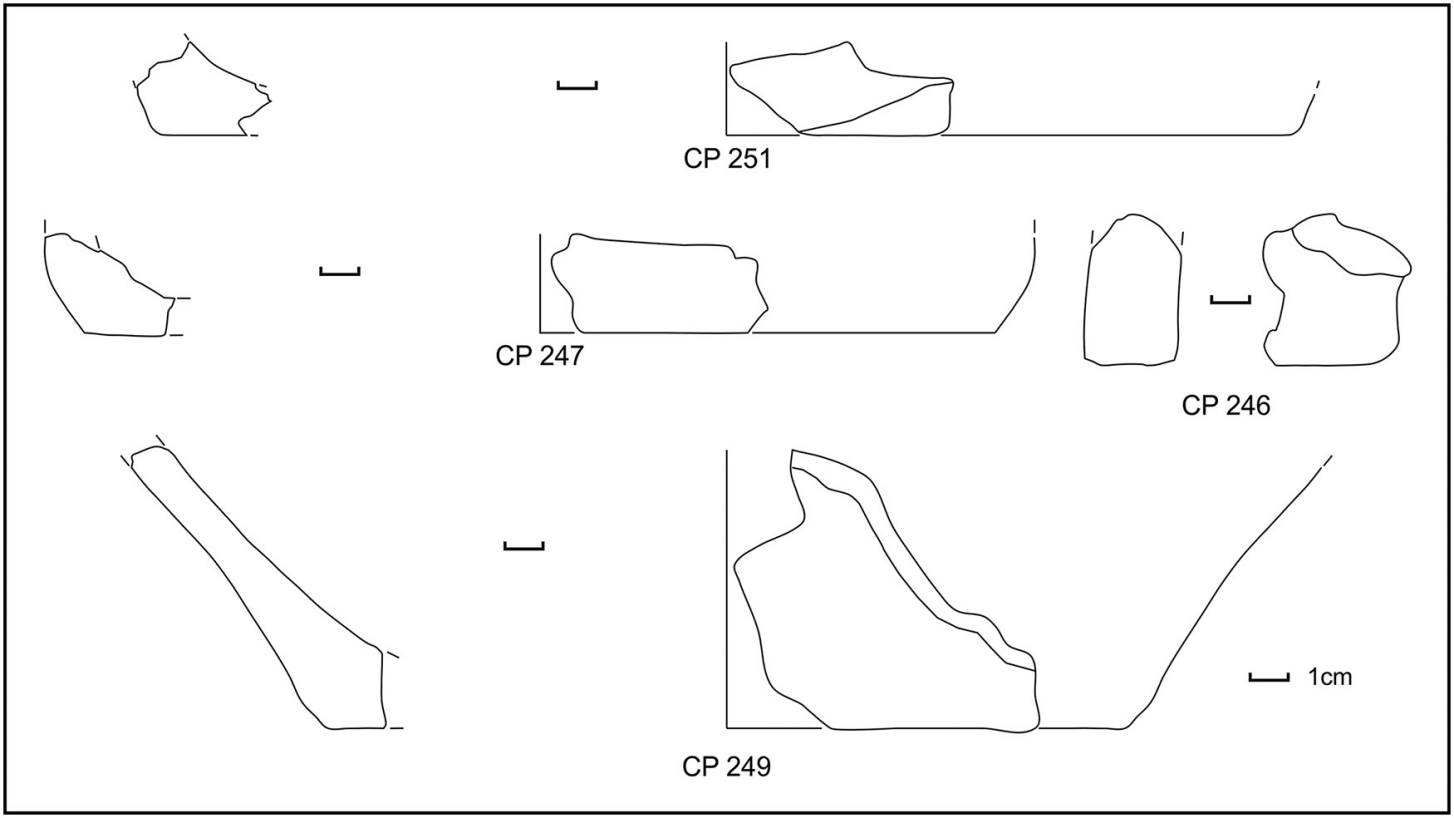
Potsherds from SU 104, the bases and a possible briquetage bar. Drawings: L. Alessandri.

The structure was covered by SU 214. Unfortunately, neither SU 217 nor SU 214 contained any diagnostic potsherd so they could only be generically dated to the Bronze Age. In both soundings D and G we were able to assess that the structures were placed just on top of the lower natural sandy layer. Su 101 and SU 217 have been interpreted as a kind of breakwater running along the ancient shoreline, to protect the soil against erosion. The stone structure SU 101 was covered by SU 104 (Fig 4), containing the typical pottery usually associated with the *briquetage* in the central Tyrrhenian Italy (Figs 15 and 16).

**Fig 17:**
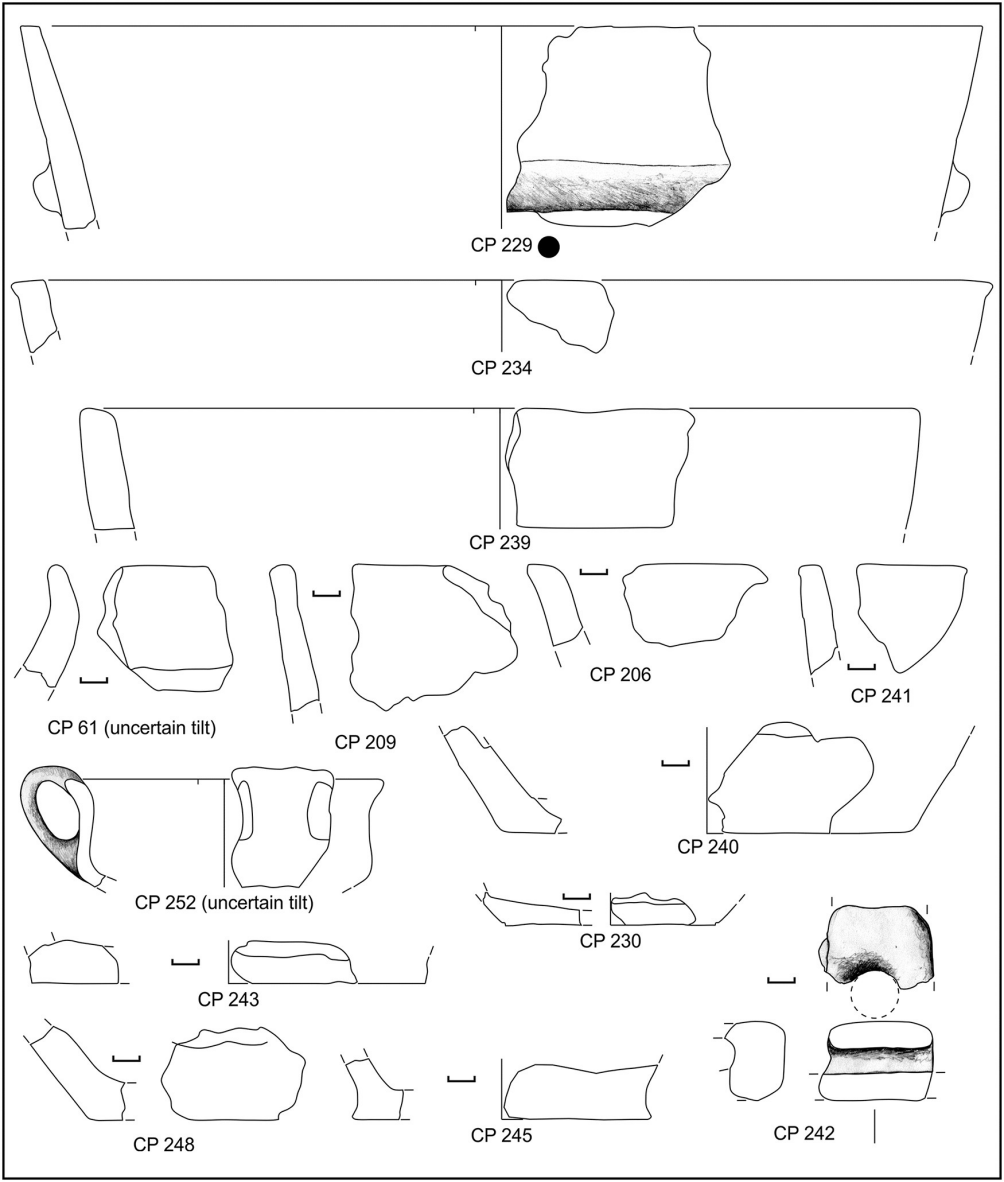
Potsherds from SU 103, open and closed vessels, bases and a possible lid. Drawings: L. Alessandri.

In SU 104, almost only half-conical reddish jars fragments were present, which cannot be precisely dated. Fortunately, the only diagnostic potsherd, a decorated fragment (CP 6), could place the layer in the MBA 3, thus a little later than the SU 202. The same pottery assemblage is also present in the more recent SU 103 (Fig 17), although mixed with a few Roman potsherds.

SU 103 can be considered as a SU 104 that was reworked in Roman times. The concentration of the potsherds in the sandy soil, in both SUs, was low (5–10%). In SU 104 the potsherds were often found clustered. No pedestals were found in the entire sounding.

**Fig 18:**
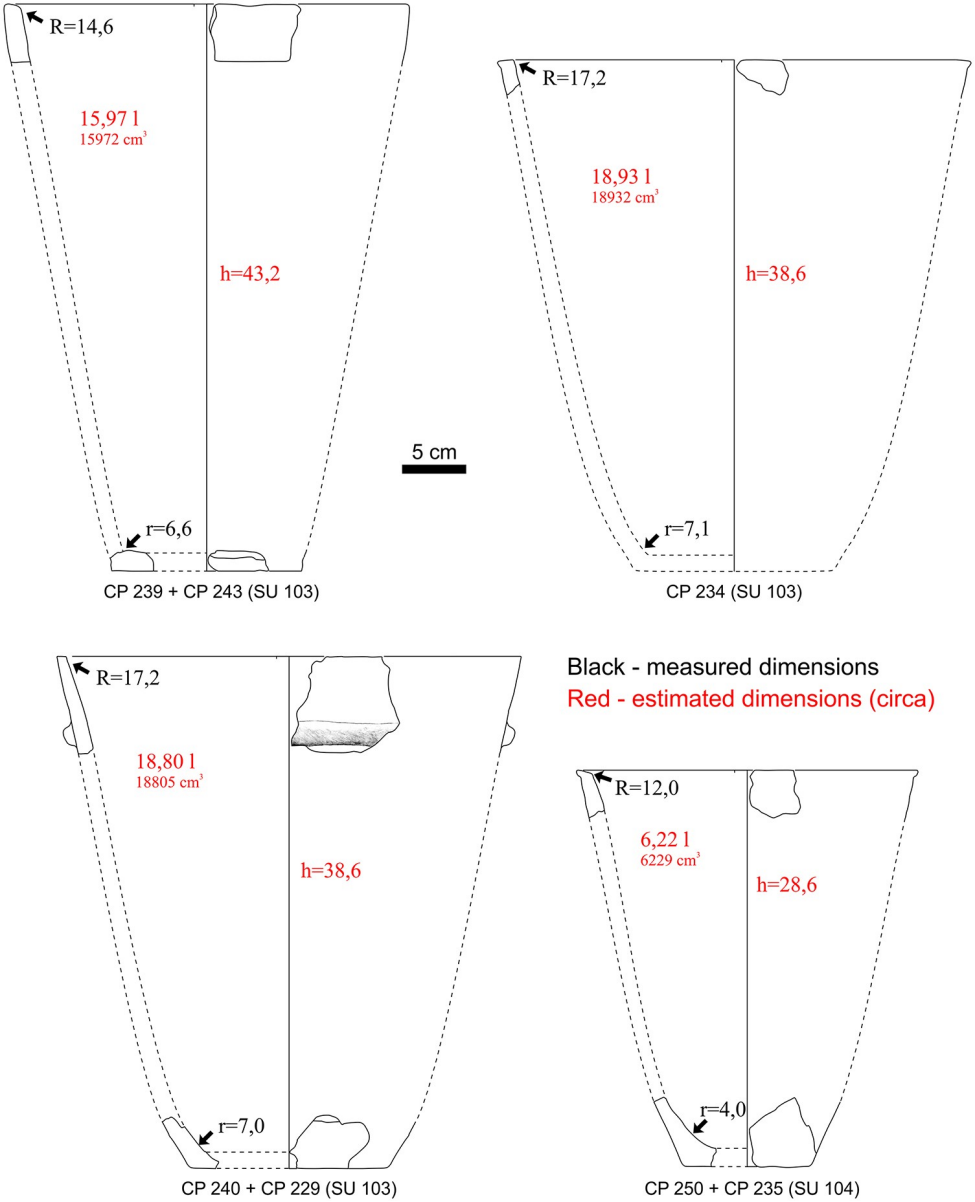
Hypothetical reconstruction of the half-conical jars from SUs 103 and 104.

A reconstruction of the half-conical jars from SU103 and 104 has been attempted only in those few cases when also the diameter could be estimated. In three cases (CP239+CP 243; CP 240+ CP 229 and CP 250+ CP 235), judging only from the thickness and inclination of the walls, a bottom and a rim could tentatively be matched (Fig 18). In the reconstruction of CP 234, we used the ratio between the diameter and the height of the CP 240 + CP 229. The vessels have an average capacity of around 15 litres (14.78 l; max 19.93 l, min 6.22 l) up to the rim.

**Fig 19:**
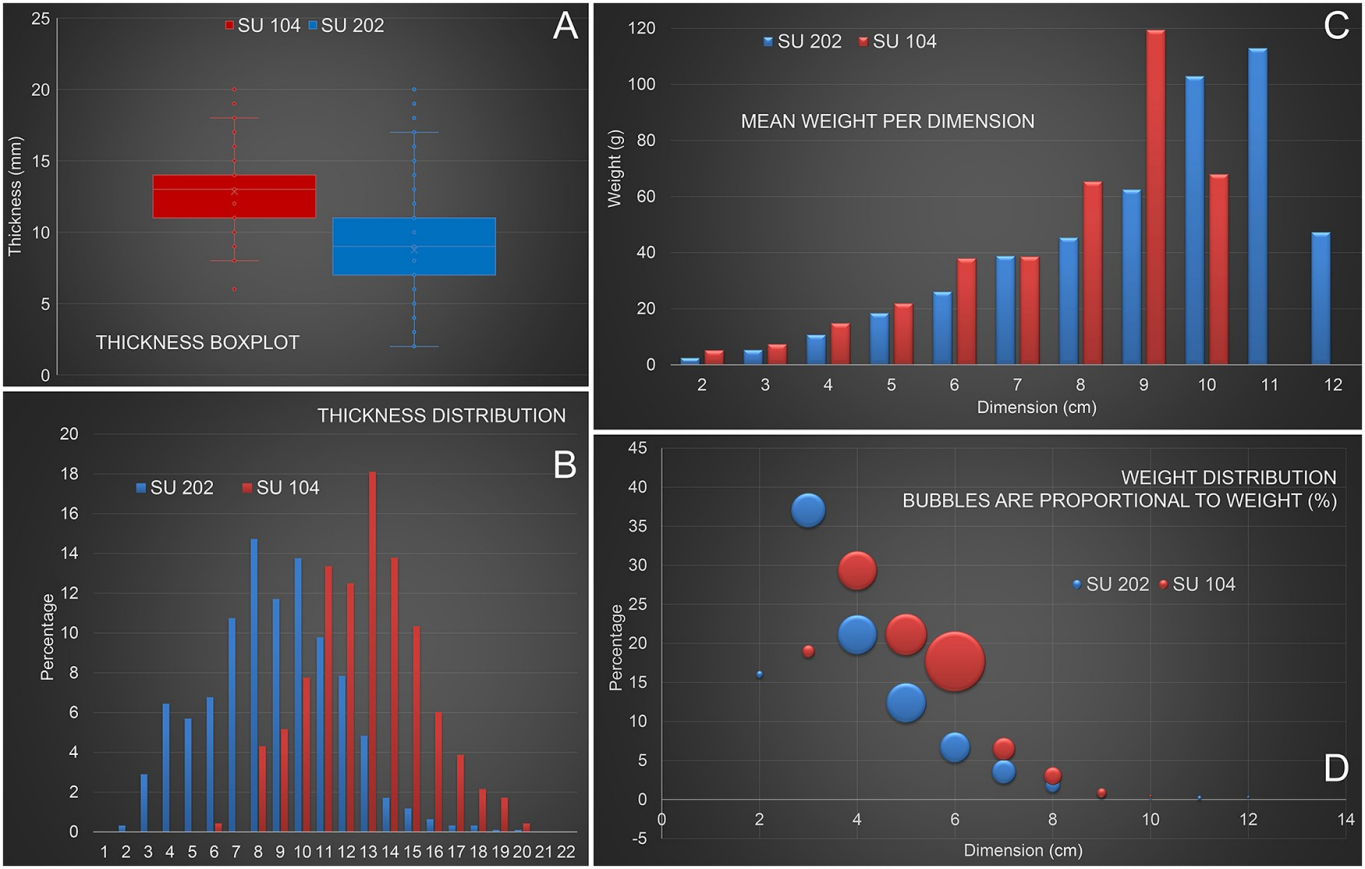
Pottery assemblages in SUs 202 and 104: Results of the statistical analyses.

Since SU 202 and SU 104 both show evidence of specialized activities, we wanted to compare the two assemblages to check for differences or similarities [154,155]. To obtain a rough estimate of their composition, we measured the weight (grouped per dimension), dimension and thickness of all the collected potsherds (including the walls) (S4 Appendix). The last parameter has proven to be the most useful. The distributions of the thickness show that in SU 202 the overall range is broader, from 2 to 20mm, and the interquartile range (IQR) spans from 7 to 11mm (median 9mm). Differently, in SU 104 the IQR spans from 11 to 15mm (median 13mm) and there are no values below 8mm except one (Fig 19A and 19B). Furthermore, the mean weight values of the potsherds per dimension in SU 104 are always higher than the ones in SU 202, with the exception of the dimensional class 10, which however only comprises one potsherd. The weight distribution shows different trends as well, with the weight percentage of SU 104 concentrated between dimensional classes 4 and 6 and a broader distribution in SU 202 (Fig 19C and 19D). All these data would confirm a strong predominance of storage vessels in SU 104, which typically have thicker walls, and an almost total absence of tableware. A simple Student’s *t* test shows that the probability that the observed difference in the thickness distribution is just a consequence of the vagaries of sampling is less than 0.1% (t = 19.4607). This evidence suggests that the specialised activities took place in two different environments and would confirm the interpretation as domestic (SU 202) and non-domestic (SU 104) contexts, respectively.

### 4.3 Archaeometry

We analysed the mineral composition and phases on inner sections of ceramics fragments by using a scanning electron microscope (SEM) equipped with energy dispersive spectrometer (EDS) (S11 Appendix). Overall, the ceramic matrix grain size in all analysed samples ranges from clay to silt size. Sub rounded inclusions of quartz, feldspars and subordinate accessory minerals/components (apatite and Fe and Mn oxides) constitute the inclusions. Abundant sand-sized (100–500 μm) quartz grains are included in the matrix, whose amount and size vary. Truly sporadic wood ash and metal micro fragments made up of a Pb-bronze alloy are also present. The relatively large quartz and K-feldspar grains have sub rounded shapes and their presence strongly suggests the use of coastal sediments as such, without evidence of fresh grain fracturing or crushing to fabricate the paste-blend.

**Fig 20:**
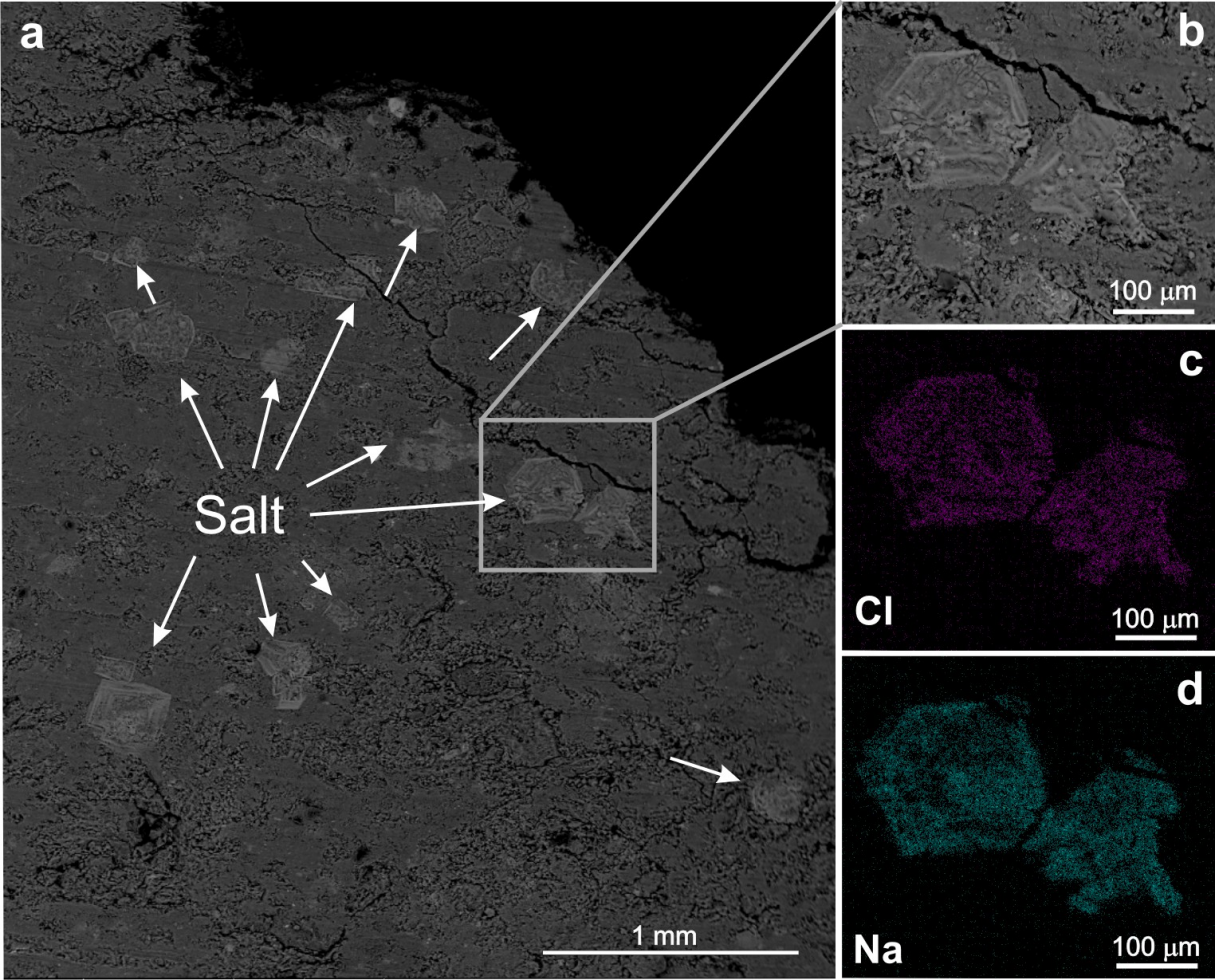
SEM-EDS images of the CP131 potsherd. Salt crystals grown within pottery microcracks took platy habit elongated perpendicularly to the vessel surfaces. (a) Details of platy crystals grown within microcracks with octahedron forms. (b) Detail of picture a with a salt crystal. (c, d) elemental maps showing the distribution of Na and Cl in the salt crystal.

Walls and interior surfaces of presumed briquetage pottery were also analysed with EMPA and SEM- EDS to establish the salinity pattern in terms of 1-D and 2-D distributions of NaCl. In CP 134, EMPA analyses evidenced that 1-D concentrations of Na show a clear gradient with lower concentrations toward the exterior of the sherd. Chlorine 1-D concentration–as evidenced along the profiles with steps of ~100 μm and analytical spots ~10 μm in diameter–show clearer lateral trends, relative to more scattered Na profile concentrations. These slightly different trends could be related to the presence of nanometric Na-bearing mineral phases (e.g., feldspars and/or Na-bearing clay minerals), in addition to sodium chloride, within the paste matrix. Due to the presence of different Na-bearing minute mineral phases (i.e., Na:Cl occurs in non-stoichiometric concentrations along 1-D profiles), to instrumental limitations (EMPA analyses averaged over spots ~10 μm in diameter)—and differently from Flad et al., [77]—we obtained no clear and conclusive evidences from 1-D profiles (not reported) in terms of lateral variations of NaCl concentrations. For these reasons, we needed to investigate in much greater detail how NaCl phases are distributed (i.e. crystal morphologies and sizes and/or amorphous forms, concentration patterns) within pottery shards. To do this, we performed elemental 2-D mapping by SEM-EDS analyses on the potsherds inner surfaces by comparing element contents for two different types of sample surfaces: a) surfaces obtained by dry cutting and polishing slices of pottery normally to the surface of vessels and b) surfaces obtained by separating samples along pre-existing microcracks into two halves, again normally to the surface of vessels. Interestingly, in CP 131 we found the largest NaCl microcrystals, up to a size of 0.3–0.4 mm, within microcracks (Fig 20).

**Fig 21:**
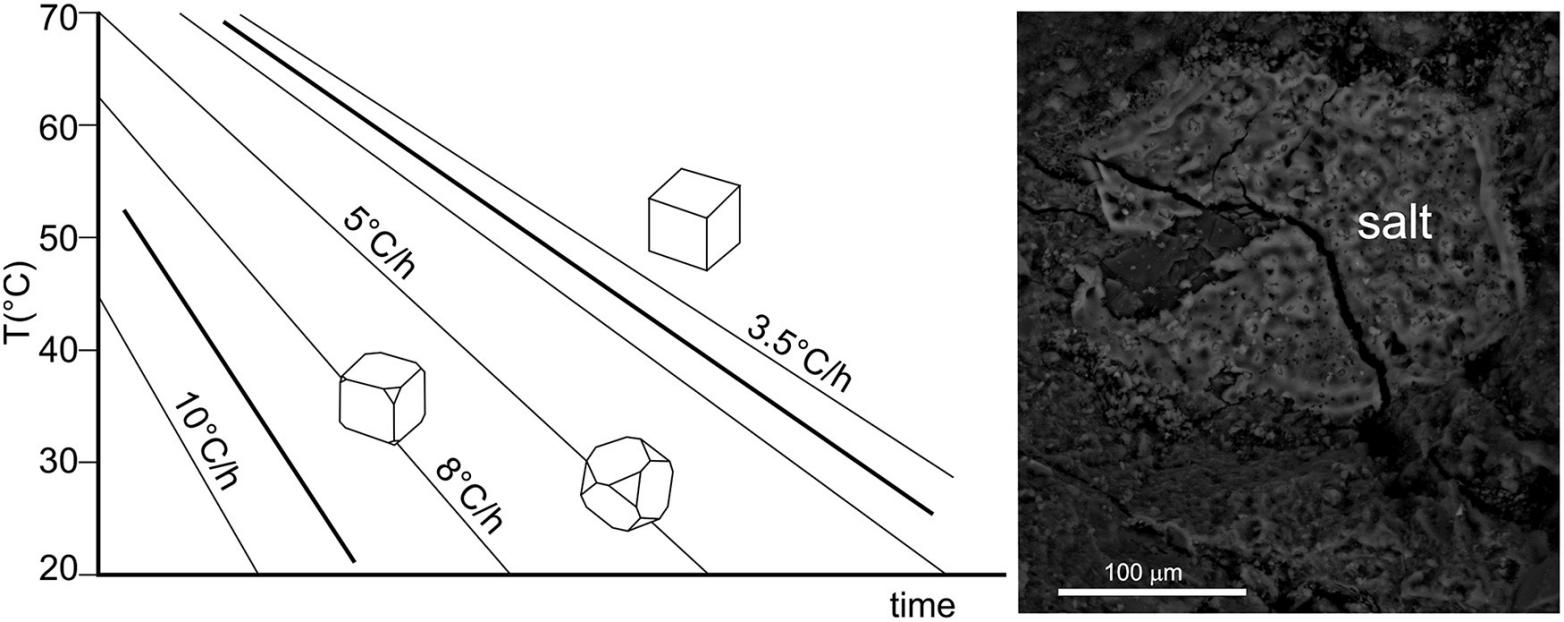
Morphology of NaCl crystals at different cooling rates for an initial saturation temperature of 95°C, graph data from [156].

As the morphology of NaCl crystals is controlled by the degree of supersaturation of the salt solution and/or by the cooling rates, the occurrence or coexistence of different NaCl phases (amorphous, cubic or octahedral crystals) provides information on the solidification/crystallization conditions [156]. In fact, the NaCl crystallization diagram (Fig 21) can be divided into three main domains. Only the NaCl cubic form {100} can exist at relatively low cooling rates (~3.5°C/h). For higher cooling rates (between 8° and 5°C/h) both {100} and {111} forms coexist with the octahedron tending to prevail for temperature decreases lower than 5°C/h. Only very small and scattered NaCl crystals can form at higher cooling rates.

The lack of pure NaCl cubic form {100} and the dominant platy habit of NaCl crystals within microcracks allow us to exclude the presence of salt within the pottery assemblage before firing. We suggest that the precipitation of NaCl during the process of brine boiling favoured the formation of microcracks within the pottery vessel; this led to different rates of Na and Cl diffusion (i.e. relatively faster within microcracks than within the ceramic matrix), thus producing different gradients of Na and Cl concentration and different growth rates of NaCl crystals. Specifically, from the NaCl habits, we distinguished two main stages of crystal growth: 1) an early stage, recorded by the occurrence of amorphous NaCl within the inner portions of NaCl aggregates, with abundant microvesicules and impurities, (Fig 21) indicates the growth of amorphous salt by a rapid cooling of NaCl supersaturated solution from a temperature very close to the water boiling point. 2) a second stage, characterised by the relatively slower cooling rate (~8°-5° C/h), as recorded by the NaCl crystals with cubic {100} plus octahedron {111} habitus in the outer portion of NaCl aggregates. Interestingly, no NaCl cubic {100} crystals of observable sizes under usual SEM magnification were detected. Since they form at cooling rates ≤ 3.5° C/h (including constant ambient temperature), this might suggest the separation or the complete evaporation of the NaCl supersaturated solution or, alternatively, a cooling time too short to allow the cubic crystals to reach observable sizes. Finally, the habitus of the detected NaCl crystals cannot be considered a possible post-depositional phenomenon, since the precipitation of salt crystals in a saline lagoon environment (i.e., within the low-gradient temperature field of Fig 21) would have produced NaCl crystals with cubic form.

## 5. Discussion

On the basis of the recorded data we will put forward some hypotheses about the jar content(s) and use(s) and afterwards we will discuss the organization of production and the Italian Bronze Age framework.

### 5.1 Content and use

The chemical evidence in CP 131 (SU 202) suggests that the analysed pottery experienced a very specific sequence of events: a NaCl supersaturated solution reached a temperature around the water boiling point, a very rapid cooling rate then occurred, followed by a slower cooling rate between 8°-5° C/h. Since there are no traces of even slower cooling rate, in terms of NaCl crystal nucleation and growth, it is possible that the content of the vessel was taken out before it could reach the ambient temperature (or that the liquid completely evaporated during the cooking). This sequence can be partly found in both the briquetage techniques and in the quick method to obtain *liquamen*, which is a fish sauce, as it is described in the Geoponica (Table 1).

**Table 1:** The subsequent reconstructed steps in the *briquetage* technique and in the quick method to obtain *liquamen*.

Stage	Salt production (*briquetage*)	*Liquamen* production (Geoponica)
NaCl supersaturated solution at about 100°	Brine boiling and evaporation	Brine, fish and oregano are boiled
Very rapid cooling rate	The fire’s out	
Slower cooling rate	The temperature of both the jar and the content gradually decrease	
Content is taken out	The jar is broken to extract the salt	The liquid is poured through a filter

However, the CP 131 preserved shape, with the out-curved rim, is typical of a container designed to pour liquid, which would be a useless feature in the briquetage technique [157]. Moreover, the overall shape, with the maximum diameter of the body greater than the mouth, it is not well suited for the evaporation. Finally, the mouth diameter limits accessibility but also increases heating effectiveness [158]. Overall, for CP 131 the stylistic evidence suggests a use in some food processing activity (cooking), which would be consistent with the *liquamen* hypothesis, but this is not explicitly confirmed by the archaeometric analysis.

In the same context, the abundant presence of pedestals, which are usually found in *briquetage* contexts, has no easy explanation, since no other typical features of the *briquetage* technique have been found. However, we are quite confident that they can be interpreted as residuals of a specialised activity, very likely connected to salt production, because of both the quantity and the rarity of such features (these are the only pedestals found in central-southern Italy to date). SU 212, which is basically a reworked SU 202, shows the same ceramic assemblage, with pedestals as well.

It must be noted that, at the moment, we have no evidence for the hypothesis that CP 131 and the pedestals have been used in the same activity.

In the sounding G a completely different situation has been unearthed. Here, the ceramic assemblages of both SU 103 and 104, almost entirely made of half-conical vessels, undoubtedly point to a specialised activity. The half-conical shape is traditionally associated with transformation/production activities. Very recently, Tencariu [159] observed an adaptive convergence in *briquetage* vessels from all over the world towards the conical and half-conical (quasi-conical in the Tencariu’s paper) shape, which he explains with similar functional constraints. In fact, those shapes ensure easy access to the interior of the vessel (during the evaporation process brine was continuously added) and uniform exposure to the heat. Furthermore, the presence of only three lugs and no handles (on 232 potsherds) in SU 104 suggests that the large majority of vessels were not meant to be transported. However, the jar weights at full (water) capacity are well below the maximum weight a person can easily carry alone, so the vessels could be manipulated quite easily [157]. Finally, only flat bottoms are present in the ceramic assemblage, which makes the use of pedestals unnecessary, at least for improving stability, and, in fact, none of them has been found. Also, the environmental conditions, with the presence of a shallow brackish or truly saline marsh or, later on, of a deeper lagoon with still saline conditions, are extremely favourable to the salt-production. So, even if in this regard the chemical analyses did not yield any conclusive result, we suggest that SU 104 and partially SU 103 are the debris of a *briquetage* activity, which took place in the immediate surroundings of sounding G. We also noted that the half-conical jars show the same reddish colour (both internal and external surfaces) of the CP 6 decorated bowl (S12 Appendix). This evidence once again suggests that this hue should be interpreted as a result of the production processes, since in all likelihood the bowl was not involved in the specialised activity.

### 5.2 Organization of production

In this discussion, the attention is focused on three of the four parameters that Costin [160] defined: context, scale, and intensity. The fourth parameter, concentration, deals with the geographical organisation of the production, but we have no new data on that subject. Costin describes the scale as the composition of the production unit which encompasses the size and the principles of labour recruitment [160]. One way to infer the scale is to measure the output. A small output would easily rule out large-scale production, while a large output can be ambiguous since, for example, it may be produced by many small households or few large workshops or, from a different point of view, many short-lasting or few long-lasting activities. In the case of SU 103 and 104, both the low percentage (5–10%) and the clustering of the jar potsherds scattered through the layers would point to small-scale production, repeated over time. Furthermore, as hypothesised before, SU 103 and SU 104 might well be the debris of a *briquetage* activity. However, in the European *briquetage* ateliers [2], and also in some Italian sites, like Duna Feniglia [161] and Torre S. Marco [162], the potsherds resulting from the deliberate breaking of the vessels were usually found very near (and interspersed among) the core of the production site, which usually comprises pits, possibly for a first solar evaporation, and kilns to boil the brine. In the underwater collection points, in the smaller island (Fig 3), almost only reddish jar potsherds have been found in points 84 and 69, closer to sounding G, and evidence for domestic environments, specifically MBA common use vessels, only come from points 29, 65 and 71. On the basis of these data, it is possible to hypothesise that the southeast portion of the small island was exclusively devoted to *briquetage* activity. This evidence, the small-scale output which took place in a specific, nondomestic area, is usually interpreted as non-kin-based, workshop production [163,164]. As for the intensity (full-time or part-time), the activities were likely to occur only during the dry season, when the temperature is higher, facilitating the natural evaporation of brine, and the rainfall is scarce.

As to the household context of SU 202 (and partly 212), CP 131 can fit in a daily domestic (non-specialised) cooking activity, while the pedestal evidence would point to individual artisans or a small kin-based work group. Unfortunately, no further speculations can be made about intensity, due to the scarce evidence but, considering the domestic environment, it is probable that this activity was carried on a part-time basis.

Trying to ascertain the context of production, which is the relationship of the productive activities and the artisans with the socio-political framework [160], is challenging. This parameter is usually seen as a *continuum* between independent specialists, whose production is determined by consumers and competing economic priorities, and attached specialists, who are full or in part controlled by the institutions or by the elites [165,166]. The independent artisans are often linked to domestic architecture, especially in small-scale productions, while the attached specialists tend to reside near the elite structures [167]. However, although this approach is a useful perspective, its archaeological identification has never been easy [168,169]. In our case, following the aforementioned line of reasoning, the layers SU 202 and partially the SU 212 would point to independent specialists while SU 103 and 104 might be placed somewhere in the *continuum*, but closer to the attached specialists.

Synthesizing, we identified two specialised activities: a possible individual artisan or small kin-based work group, in a domestic area, probably part-time, in SU 202 (and partly 212); a *briquetage*, non-kin-based, workshop production, in a nondomestic area, probably part-time, in SUs 104 and 103.

### 5.3 The Bronze Age socio-political framework

**Fig 22:**
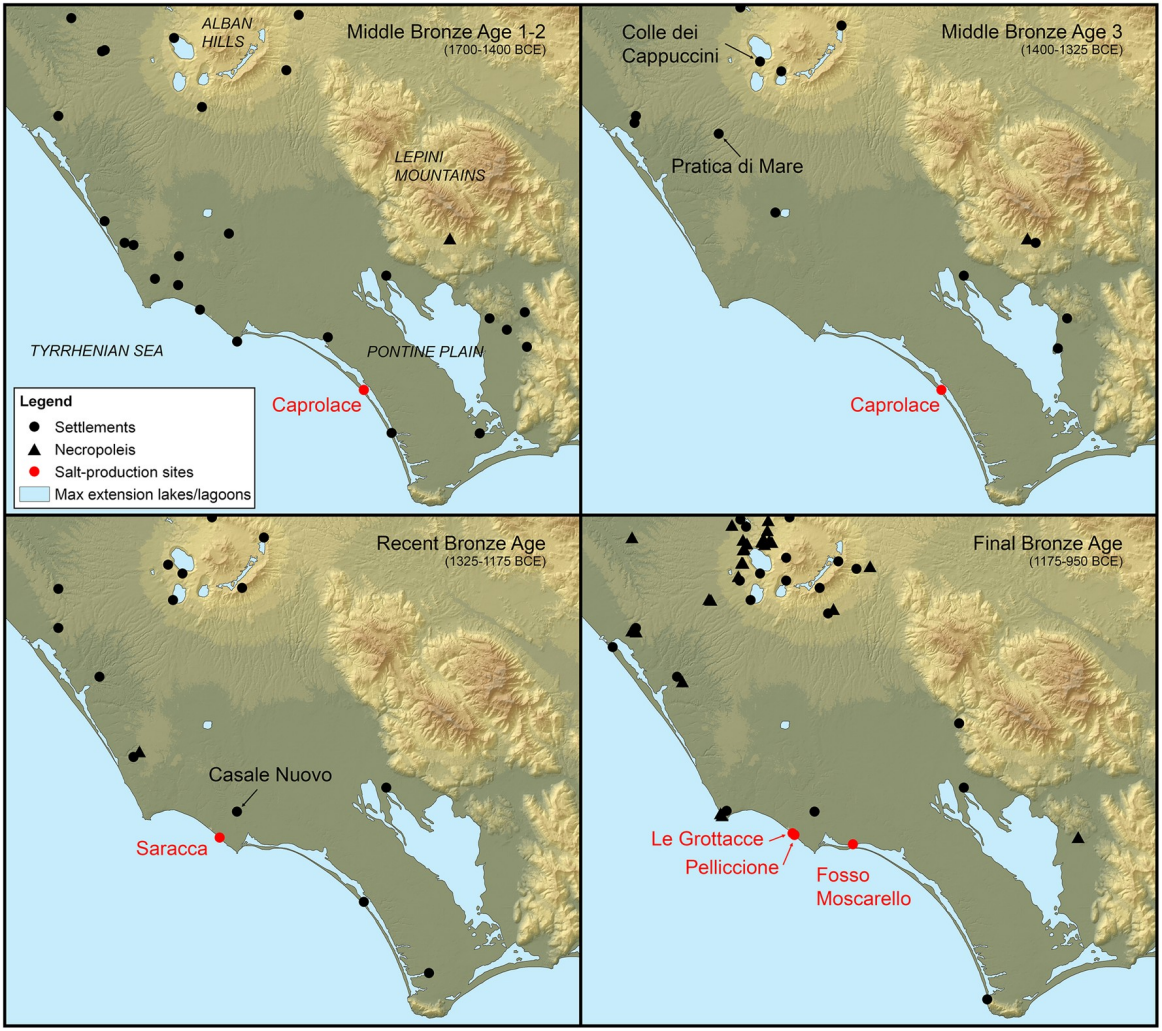
Development of settlements and necropoleis in southern *Latium* from MBA to FBA. Data from [175]. Map derived from Shuttle Radar Topography Mission elevation datasets (SRTM NASA V3).

In central Tyrrhenian Italy, the Bronze Age is the period in which the small, egalitarian, horticultural societies develop in much bigger chiefdom and early states. The trajectory to these more complex polities was not easy nor straightforward, with local and significant differences ([4,170–175], all with different nuances). This substantial socio-political shift coincides, all over Italy, with some clear trends in settlement patterns: *stabilization*, which indicates a progressively longer life of the villages, and *selection and concentration*, which indicate a decrease in the number of settlements together with an increase of their average dimensions [3,176]. At the beginning of the Bronze Age, southern *Latium* is generally characterised by small (1–2 ha) dispersed settlements (Fig 22).

The latter most likely undergo regular short-distance migrations, probably in search for fresh terrains [175,177]. Inter-polities conflicts appear to be absent since very few settlements show tactical and/or strategical potential. This picture can be easily traced until the MBA 1–2. However, in the next phase (MBA 3), the settlements life-span gradually starts to increase (more consecutive subphases) and some of them clearly favour naturally fortified locations, like Pratica di Mare [178], Ficana [179] and Colle dei Cappuccini [180], marking the start of the two aforementioned trends [175]. In the subsequent phase, the Recent Bronze Age (RBA), the first Mycenaean and Italo-Mycenaean pottery appears in *Latium*, from Casale Nuovo [181], which indicates a wide-ranging network of exchanges and the presence of people interested in highly symbolic artefacts. Moreover, the funerary practice shifts from collective inhumations in natural caves [182–185], which underline a substantially egalitarian conception of the death, to individual incinerations in open terrain only for selected individuals [186,187]. Later on, in the FBA, these burials were also characterised by miniature vessels [188–190] and some infant tombs held exceptional grave gifts [191]. This evidence shows that, in all likelihood, the appearance of the elites and the concomitant institutionalization of power (with ascribed status) is part of a progressive phenomenon which has its roots in the MBA 3 and further develops until the FBA [175,192]. However, already in 1996, R. Peroni proposed a generic socio-economic framework for the development of the Bronze Age Italian polities which has been widely adopted in the Italian debate [170,193]. As for Central Italy, he proposed, for the late Bronze Age (which encompasses both RBA and FBA), the existence of communities based on patron-client relationships. In his opinion, in a Bronze Age scenario still characterised by the collective ownership of land, the elites (the patrons) would have ascertained their full economic potential by controlling “marginal” resources (e.g. metal trade, exchange networks of luxury items, and storable goods). The aim of this strategy would have been to create relationships of dependence with the (non-kin) clients. Actually, our reconstructed organization of production unit in SUs 104 and 103, a non-kin-based workshop in a separate area of the settlement, would fit very well into this framework: a *briquetage* workshop ruled by an emerging elite and run by its (attached) clients.

## 6. Conclusion

The multidisciplinary study based on the careful morphological and stylistic analyses of the potsherds, the archaeometric studies and the environmental reconstruction, proved to be a strong approach in defining the content and use of the vessels being investigated. The presence of a possible *briquetage* site in southern *Latium* had already been hypothesised by Alessandri [64], and Nijboer and colleagues [63]. However, at that moment, strong evidence was lacking and the chronologies suggested a more recent start (in the RBA) of the activity. The Caprolace data has enabled us to shift the latter to the MBA 3, in concomitance and possible connection with the first emerging elite. Moreover, the almost exclusive presence, in the SU 103 and 104, of one type of vessel (half-conical jar) forms new and interesting evidence which also suggests that the usual Italic *briquetage* assemblages, which are composed of many different shapes (see paragraph 2.3, point 2) should be reinterpreted, like for example the ones from Pelliccione. In the latter, already interpreted as a salt-making site [63], tens of different types of vessels have been found, including our half-conical vessel. We suggest that the possible alternative uses of the non-half-conical vessels should be explored by archaeometric and stylistic analyses. In this regard, our analyses suggest that the “fish hypothesis” might be a viable line of research. Finally, from a mere methodological point of view, we stress the importance of sample preparation and the key role of cross comparing the results from different analytical methods (e.g., 1-D and 2-D elemental distributions by SEM-EDS and EMPA) and experimental data on the effects of temperature gradients on NaCl crystal morphologies and growth rates from aqueous solutions in archaeometric studies of suspected *briquetage* materials. Sample preparations should include the dry-cutting of different types of analysable areas including both thin polished and along-microcracks surfaces perpendicular to the surface of vessels, in order to preserve the salt crystals which may be present.

## Supporting information

S1 Appendix Chronology and references of the sites illustrated in Fig 1. (DOCX)

S2 Appendix Map of the Italian sites with pottery parallels. Number of sites in S3 Appendix. Aerial photo from Esri, Digital Globe, GeoEye, Earthstar Geographics, CNES/Airbus DS, USDA, USGS, AeroGRID, IGN, and the GIS User Community.
(TIF)

S3 Appendix Parallels and chronologies of the Caprolace potsherds. (DOCX)

S4 Appendix Data and statistics about the SU 202 and 104 potsherds. (XLSX)

S5 Appendix Radiocarbon dates from Caprolace. (XLSX)

S6 Appendix Diatoms data. (XLSX)

S7 Appendix Photo of the exposed profile of the sounding F before the excavation. Photo by M. F. Rolfo.
(TIF)

S8 Appendix The sounding F exposed profile and the surface of SU 202. Drawings: K. Achino, A. Fiorillo, A. Cesaretti.
(TIF)

S9 Appendix The locations of the corings. Drawings: L. Alessandri.
(TIF)

S10 Appendix The descriptions of the corings. (XLSX)

S11 Appendix List of the analysed potsherds (EMPA and SEM-EDS). (XLSX)

S12 Appendix Pictures of potsherds from SUs 103 and 104. Photo by M. F. Rolfo and A. Ferracci.
(TIF)
