# Supplementary material for: Salt or fish (or salted fish)? The Bronze Age specialised sites along the Tyrrhenian coast of Central Italy: New insights from Caprolace settlement
Source: PLoS One. 2019 Nov 13;14(11):e0224435. doi: 10.1371/journal.pone.0224435 (PMC6853298; doi:10.1371/journal.pone.0224435)
Supplement: S1 Appendix — (DOCX) [file pone.0224435.s001.docx]

| **N.** | **Site** | **Chronology** | **References** |
| --- | --- | --- | --- |
| 1 | Isola di Coltano | MBA1-2 to FBA | [1–3] |
| 2 | Galafone | FBA?, EIA | [4] |
| 3 | Riva degli Etruschi | FBA | [5–7] |
| 4 | Poggio del Molino | RBA, FBA | [8–10] |
| 5 | La Torraccia | FBA3 or EIA | [5,11] |
| 6 | Baratti | FBA or EIA | [12,13] |
| 7 | Torre Mozza | FBA or EIA | [7,14,15] |
| 8 | Puntone Nuovo-Le Chiarine | FBA | [16–19] |
| 9 | Puntone Nuovo-Campo da Gioco | EIA | [20] |
| 10 | Puntone Nuovo-Meleta | EIA | [15,21–23] |
| 11 | Puntone Nuovo-Fiumara | MBA, FBA | [24] |
| 12 | Puntone Nuovo-Fosso del Fico | FBA | [15,25] |
| 13 | Portiglioni - Campo da Gioco | FBA3 or EIA | [15,22,23,26,27] |
| 14 | Portiglioni | ? | [28] |
| 15 | Poggio Carpineta | ? | [15] |
| 16 | Tombolello | FBA or EIA | [29–31] |
| 17 | Casa San Giuseppe | EIA | [29–31] |
| 18 | Punta degli Stretti | FBA3 | [32–36] |
| 19 | Poggio Pertuso | EIA | [31,37–39] |
| 20 | Duna Feniglia | EIA | [40–44] |
| 21 | Fontanile delle Serpi | FBA or EIA | [45–48] |
| 22 | Le Saline di Tarquinia | EIA | [49,50] |
| 23 | Bagni Sant’Agostino | EIA | [51] |
| 24 | La Frasca | EIA | [52–54] |
| 25 | Acque Fresche | EIA | [52,55] |
| 26 | Torre Valdaliga | EIA | [52,56–58] |
| 27 | La Mattonara | FBA?, EIA | [52,57,59–61] |
| 28 | Punta del Pecoraro | EIA | [62,63] |
| 29 | Malpasso | EIA | [64,65] |
| 30 | Marangone | EIA | [59,64,66–68] |
| 31 | Torre Chiaruccia/Foce Guardiole | EIA | [52,64] |
| 32 | Colonia dei Calabresi | EIA | [69] n. 47 |
| 33 | Quartaccia | FBA?, EIA | [70] |
| 34 | Grottini | EIA | [57,70] |
| 35 | Greppa della Macchiozza | FBA or EIA | [46,48] |
| 36 | Cretarossa/San Rocco (also known as Nettuno Depuratore) | EIA | [71–73] |
| 37 | Le Grottacce | FBA | [72,74–76] |
| 38 | Pelliccione | FBA | [72,74–78] |
| 39 | Saracca | RBA | [72,74,76,79] |
| 40 | Area Stop 4 | EBA?, MBA?, FBA? | [72,74,80,81] |
| 41 | Fosso Moscarello | FBA | [72,74,76] |
| 42 | Caprolace | RBA or FBA | [72,74] |
| 43 | La Cotarda | FBA or EIA | Unpublished |

# Chronology and references of the sites illustrated in Fig 1

## Chronology and references

## Simplified chronological scheme of Italy

Absolute chronologies from [48,82,83]

| Acronym | Phases and subphases | Absolute chronology (all y. BCE) |
| --- | --- | --- |
| EBA | Early Bronze Age | 2000-1700 |
| MBA1-2 | Middle Bronze Age subphases 1 and 2 | 1700-1400 |
| MBA3 | Middle Bronze Age subphase 3 | 1400-1300 |
| RBA | Recent Bronze Age | 1300-1150 |
| FBA1-2 | Final Bronze Age subphases 1and 2 | 1150-1050 |
| FBA3 | Final Bronze Age subphase 3 | 1050-950 |
| EIA | Early Iron Age | 950-725 |

# References

1. Di Fraia T, Secoli L. Il sito dell’età del Bronzo di Isola di Coltano. In: Negroni Catacchio N, editor. Atti del V incontro di studi Preistoria e Protostoria in Etruria Paesaggi d’acque. 2002. pp. 79–93.

2. Pasquinucci M, Menchelli S. The Isola di Coltano Bronze Age village and the salt production in North coastal Tuscany (Italy). In: Weller O, editor. Archéologie du sel: techniques and sociétés Colloque 122, XIVe congrés UISPP. 2002. pp. 177–188.

3. Pasquinucci M, Menchelli S. Isola di Coltano (Coltano)-Pisa. In: Zanini A, editor. Dal Bronzo al Ferro Il II millennio aC nella Toscana centro-occidentale. 1997. pp. 49–53.

4. Pasquinucci M, Del Rio A, Menchelli S. Terra e acque nell’Etruria nord-occidentale. In: Negroni Catacchio N, editor. Atti del V incontro di studi Preistoria e Protostoria in Etruria Paesaggi d’acque. 2002. pp. 51–61.

5. Fedeli F. Populonia. Storia e territorio. Firenze; 1983.

6. Fedeli F. Riva degli Etruschi (San Vincenzo-LI). In: Zanini A, editor. Dal Bronzo al Ferro Il II millennio aC nella Toscana centro-occidentale. 1997. pp. 123–124.

7. Fedeli F, Galiberti A. Insediamenti dell’età del Bronzo nel comprensorio di Piombino (Livorno). Nota Preliminare. Rass di Archeol. 1979;1–2: 147–238.

8. Fedeli F. Poggio del Molino (Piombino-LI). In: Zanini A, editor. Dal Bronzo al Ferro Il II millennio aC nella Toscana centro-occidentale. Pisa; 1997. pp. 129–134.

9. Fedeli F, Franchi R, Pallecchi P. Poggio del Molino. In: Martini F, Pallecchi P, Sarti L, editors. La ceramica preistorica in Toscana Artigianati e materie prime dal Neolitico all’età del Bronzo. Città di Castello; 1996. pp. 218–223.

10. Fedeli F. Populonia, storia e territorio. Firenze: All’Insegna del Giglio; 1983.

11. Fedeli F. La Torraccia (San Vincenzo-LI). In: Zanini A, editor. Dal Bronzo al Ferro Il II millennio aC nella Toscana centro-occidentale. Pisa; 1997. p. 125.

12. Baratti G. Un sito per la produzione del sale sulla spiaggia di Baratti (area Centro Velico) alla fine dell’età del Bronzo. Materiali per Populonia, 9. 2010. pp. 243–260.

13. Fedeli F. Golfo di Baratti (Piombino-LI). Spiaggia antistante la pineta del Casone. In: Zanini A, editor. Dal bronzo al ferro Il II millennio aC nella Toscana centro-occidentale. Pisa; 1997. pp. 127–129.

14. Fedeli F. Torre Mozza (Piombino-LI). In: Zanini A, editor. Dal Bronzo al Ferro Il II millennio aC nella Toscana centro-occidentale. Pisa; 1997. p. 125.

15. Aranguren BM. Il Golfo di Follonica in età protostorica: l’idrografia antica e i sistemi insediamentali. Atti del V incontro di studi Preistoria e Protostoria in Etruria Paesaggi d’acque. 2002. pp. 111–122.

16. Aranguren BM, Burchianti F. Scarlino (GR). Località Le Chiarine: lo scavo della fornace A. Not della Soprintend per i Beni Archeol della Toscana. 2009;4/2008: 333–335.

17. Aranguren BM. Una fornace per ceramica dell’età del bronzo finale in località Le Chiarine, Puntone Nuovo, Scarlino (GR). Nota preliminare. In: Negroni Catacchio N, editor. Atti dell’VIII incontro di studi preistoria e protostoria in Etruria Paesaggi reali e paesaggi mentali. Milano; 2008. pp. 593–602.

18. Aranguren BM, Burchianti F. Scarlino (GR). Località Le Chiarine: l’impianto produttivo per ceramica dell’età del Bronzo Finale. Not della Soprintend per i Beni Archeol della Toscana. 2008;3/2007: 456–459.

19. Aranguren BM, Cinquegrana MR. Siti industriali del litorale marino del Golfo di Follonica tra il Bronzo finale e il primo Ferro. Atti della L Riunione Scientifica dell’Istituto Italiano di Preistoria e Protostoria, Preistoria del Cibo. 2015. Available: www.preistoriadelcibo.it/contributi/3_39.pdf

20. Aranguren BM, Cinquegrana MR, De Bonis A, Guarino V, Morra V, Pacciarelli M. Le strutture e lo scarico di olle del Puntone Nuovo di Scarlino (GR), e i siti costieri specializzati della protostoria mediotirrenica. Riv di Sci Preist. 2014;LXIV: 227–259.

21. Aranguren BM. ll sistema insediativo del territorio di Scarlino in età protostorica. Scarlino Arte, Storia e Territorio. 2003. pp. 9–23.

22. Bartoloni G. Insediamenti della tarda età del bronzo nel distretto minerario toscano. Pact. 1988;21: 71–72.

23. Cucini C. Topografia del territorio delle valli del Pecora e dell’Alma. In: Francovich R, editor. Scarlino I Storia e territorio. Firenze; 1985. pp. 147–314.

24. Aranguren BM. Il comprensorio delle Colline Metallifere in età pre-protostorica. Atti della XXXIV Riunione Scientifica dell’Istituto Italiano di Preistoria e Protostoria, Preistoria e Protostoria della Toscana. Firenze; 2001. pp. 489–502.

25. Perazzi P. Scarlino - Puntone Nuovo. Loc. Fosso del Fico. Stud e Mater. 1991;VI: 357.

26. Aranguren BM, Castelli S. Scarlino (GR). Testimonianze di attività produttive a Portiglioni. Not della Soprintend per i Beni Archeol della Toscana. 2006;1: 293–299.

27. Aranguren BM, Castelli S. Fra mare e laguna: ipotesi interpretative per il sito produttivo di Portiglioni, Scarlino (GR). In: Lugli F, Stoppiello AA, Biagetti S, editors. Atti del 4° Convegno Nazionale di Etnoarcheologia. Oxford: BAR International Series, 2235; 2011. pp. 9–16.

28. Aranguren BM. Il Puntone di Scarlino (Grosseto): la costa. Problemi di tutela. In: Firmati M, editor. Coste e Mari della Toscana. Livorno; 2008. pp. 77–96.

29. Ciampoltrini G. Insediamenti nella bonifica di Talamone (Orbetello, Grosseto). Un contributo per l’insediamento perilagunare dell’Età del Bronzo in Toscana. Atti della XXXIV Riunione Scientifica dell’Istituto Italiano di Preistoria e Protostoria, Preistoria e Protostoria della Toscana. Pisa; 2001. pp. 533–543.

30. Ciampoltrini G, Pierfederici P. L’insediamento perilagunare da Talamone al Chiarone dall’età del Bronzo alla prima età del Ferro. Appunti per l’indagine. Atti del V incontro di studi Preistoria e Protostoria in Etruria Paesaggi d’acque. 2002. pp. 123–132.

31. Negroni Catacchio N, Cardosa M. Dalle sorgenti al mare. Rapporti tra l’area interna e le lagune costiere nel territorio tra Fiora e Albegna. In: Negroni Catacchio N, editor. Atti del V incontro di studi Preistoria e Protostoria in Etruria Paesaggi d’acque. 2002. pp. 157–177.

32. Arcangeli L, Pellegrini E, Poggesi G. L’insediamento sommerso di Punta degli Stretti nella laguna di Orbetello (Grosseto) e il popolamento dell’area costiera tra i ﬁumi Fiora e Albegna in età protostorica. Atti della XXXIV Riunione Scientifica dell’Istituto Italiano di Preistoria e Protostoria, Preistoria e Protostoria della Toscana. 2001. pp. 545–555.

33. Arcangeli L, Pellegrini E, Poggesi G. L’insediamento sommerso di Punta degli Stretti nella laguna di Orbetello (Gr). In: Negroni Catacchio N, editor. Atti del V incontro di studi Preistoria e Protostoria in Etruria Paesaggi d’acque. Milano; 2002. pp. 133–140.

34. Poesini S. La produzione ceramica di Punta degli Stretti (Orbetello, GR): aggiornamento degli studi. In: Negroni Catacchio N, editor. Atti del X incontro di studi preistoria e protostoria in Etruria L’Etruria dal Paleolitico al Primo Ferro Lo stato delle ricerche. Milano; 2012. pp. 553–566.

35. Poesini S. Il materiale di Punta degli Stretti: prospettive di studio, conservazione e fruizione. Not della Soprintend per i Beni Archeol della Toscana. 2008;3/2007: 778–780.

36. Poggesi G. Punta degli Stretti. In: Poggesi G, Rendini P, editors. Memorie sommerse Archeologia subacquea in Toscana. Pitigliano; 1998. pp. 216–222.

37. Bronson RC, Uggeri G. Isola del Giglio, Isola di Giannutri, Monte Argentario, Laguna di Orbetello. Stud Etruschi. 1970;XXXVIII: 201–214.

38. Cardosa M. La frequentazione protostorica del Tombolo di Feniglia (Orbetello - GR). In: Negroni Catacchio N, editor. Atti del V incontro di studi Preistoria e Protostoria in Etruria Paesaggi d’acque. 2002. pp. 145–155.

39. Cardosa M. “Paesaggi d’acque” al Monte Argentario. In: Negroni Catacchio N, editor. Atti del VI incontro di studi preistoria e protostoria in Etruria Miti simboli decorazioni. 2004. pp. 405–415.

40. Benedetti L, Capuzzo P, Fontana L, Rossi F. Nuovi dati dallo scavo di Duna Feniglia (Orbetello, GR). In: Negroni Catacchio N, editor. Atti del IX incontro di studi Preistoria e Protostoria in Etruria L’alba dell’Etruria Fenomeni di continuità e trasformazione nei secoli XII-VIII aC. 2010. pp. 157–167.

41. Benedetti L, Capuzzo P, Fontana L, Rossi F. Paesaggi d’acque. Duna Feniglia, loc. Ansedonia. Scavo di un insediamento del Primo Ferro: risultati e prospettive. In: Negroni Catacchio N, editor. Atti dell’VIII incontro di studi preistoria e protostoria in Etruria Paesaggi reali e paesaggi mentali. 2008. pp. 261–284.

42. Negroni Catacchio N, Cardosa M, Rossi F. Duna Feniglia (Orbetello (GR). Un insediamento villanoviano per la probabile produzione del sale. Atti della L Riunione Scientifica dell’Istituto Italiano di Preistoria e Protostoria, Preistoria del Cibo. 2015. Available: http://www.preistoriadelcibo.it/contributi/3_40.pdf

43. Rossi F. Duna Feniglia – Sede Forestale (sito TF01). Un sito produttivo villanoviano. In: Negroni Catacchio N, Cardosa M, Dolfini A, editors. Paesaggi d’Acque La Laguna di Orbetello e il Monte Argentario tra Preistoria ed Età Romana. Milano; 2017. pp. 230–251.

44. Rossi F, Campo L, Cappello I, Cardosa M, Lepri A, Luciano M. Duna Feniglia (Orbetello, GR). I risultati delle ultime campagne di scavo (2011-2012) nell’area nord-occidentale. In: Negroni Catacchio N, editor. Atti dell’XI incontro di Preistoria e Protostoria in Etruria Paesaggi cerimoniali. 2014. pp. 681–688.

45. Brunetti Nardi G. Repertorio degli scavi e delle scoperte archeologiche dell’Etruria meridionale (1971-1975). Roma; 1981.

46. di Gennaro F. Forme di insediamento tra Tevere e Fiora dal Bronzo finale al principio dell’età del Ferro. 14 B di SE, editor. Firenze; 1986.

47. Fugazzola Delpino MA, Delpino F. Il Bronzo finale nel Lazio settentrionale. Atti della XXI Riunione Scientifica dell’Istituto Italiano di Preistoria e Protostoria. Firenze; 1979. pp. 275–316.

48. Pacciarelli M. Dal villaggio alla città. La svolta protourbana del 1000 a.C. nell’Italia tirrenica. Firenze; 2001.

49. Mandolesi A. La “prima” Tarquinia: L’insediamento protostorico sulla Civita e nel territorio circostante. Firenze; 1999.

50. Mandolesi A. L’insediamento Villanoviano. Teknos. 1996;6, supp.: 35–37.

51. Pacciarelli M. Insediamento, territorio, comunità in Etruria meridionale agli esordi del processo di urbanizzazione. Sci dell’Antichità. 1991;5: 163–208.

52. Barbaranelli F. Villaggi villanoviani dell’Etruria meridionale marittima. Bull di Paletnologia Ital. 1956;65: 455–489.

53. Capuani F. Ricerche protostoriche sulla costiera a nord di Civitavecchia. Boll dell’Associazione Archeol Centumcellae. 1971; 55–68.

54. Toti O. Brevi considerazioni sulle presenze costiere della prima età del Ferro. Boll della Soc Tarquiniese di Arte e Stor. 1993;XXII: 41–66.

55. Mandolesi A, Trucco F. L’abitato costiero della prima età del ferro di Acque Fresche (Civitavecchia - RM). In: Negroni Catacchio N, editor. Atti del IV incontro di studi Preistoria e Protostoria in Etruria Paesaggi d’acque. Milano; 2002. pp. 495–503.

56. Belardelli C. Torre Valdaliga. Ferrante Rittatore Vonwiller e la Maremma, 1936-1976 Paesaggi naturali, umani, archeologici. 1999. pp. 79–90.

57. Belardelli C, Pascucci P. Il villanoviano a nord di Roma: i siti costieri del territorio di Civitavecchia. Proceedings of XIII international congress of Union Internationale des Sciences Préhistoriques et Protohistoriques, 4. 1998. pp. 408–417.

58. Maffei A. Il complesso abitativo proto-urbano di Torre Valdaliga. La preistoria e la protostoria nel territorio di Civitavecchia. Civitavecchia; 1981. pp. 96–217.

59. Barbaranelli F. Ricerche paletnologiche sulla costiera tirrenica a nord di Capo Linaro. Proceedings of VI international congress of Union Internationale des Sciences Préhistoriques et Protohistoriques Vol 3. 1966. pp. 19–23.

60. Pascucci P. La Mattonara. Ferrante Rittatore Vonwiller e la Maremma 1936-1976: paesaggi naturali, umani, archeologici, Atti del convegno. 1999. pp. 91–102.

61. Toti O. Civitavecchia - Rinvenimento di tre “pozzetti domestici” in località “La Mattonara”». Not degli Scavi di Antich. 1962;XVI: 301–310.

62. Belardelli C, Pascucci P. I siti costieri del territorio di Civitavecchia e S. Marinella nella prima età del ferro. Risultati preliminari di una revisione critica dei dati. Boll della Soc Tarquiniese di Arte e Stor. 1996;25: 343–398.

63. Radmilli AM. Attività del Museo Nazionale Preistorico ed Etnografico “L. Pigorini” - anni 1946-51. Bull di Paletnologia Ital. 1952;63: 63–80.

64. Barbaranelli F. Ricerche paletnologiche nel territorio di Civitavecchia. Gli abitati dell’età del bronzo. Bull di Paletnologia Ital. 1955;64: 382–400.

65. Peroni R. La stazione preistorica di Malpasso presso Civitavecchia. Bull di Paletnologia Ital. 1953;63: 131–146.

66. Belardelli C, Pascucci P. Lo sfruttamento delle risorse marine nell’età del ferro: il caso di Marangone (Santa Marinella, Roma). In: Negroni Catacchio N, editor. Atti del V incontro di studi Preistoria e Protostoria in Etruria Paesaggi d’acque. Milano; 2002. pp. 241–255.

67. D’Ercole V, di Gennaro F, Trucco F. Notiziario. Marangone (Santa Marinella). Riv di Sci Preist. 1996;XLVII: 441–442.

68. Trucco F, di Gennaro F, D’Ercole V. Contributo alla conoscenza della costa dell’Etruria meridionale nella protostoria. Lo scavo 1994 al Marangone (S. Marinella - RM). In: Negroni Catacchio N, editor. Atti del V incontro di studi Preistoria e Protostoria in Etruria Paesaggi d’acque. 2002. pp. 231–240.

69. Belardelli C, Angle M, di Gennaro F, Trucco F. Repertorio dei siti protostorici del Lazio. Province di Roma, Viterbo e Frosinone. Belardelli C, Angle M, di Gennaro F, Trucco F, editors. 2007.

70. Barbaranelli F. Ulteriori ricerche paletnologiche nel Civitavecchiese. Bull di Paletnologia Ital. 1959;67–68: 219–228.

71. Alessandri L, Tol GW. Cretarossa/San Rocco. In: Belardelli C, Angle M, di Gennaro F, Trucco F, editors. Repertorio dei siti protostorici del Lazio - province di Roma, Viterbo e Frosinone. 2007. pp. 215–218.

72. Alessandri L. L’occupazione costiera protostorica del Lazio centromeridionale. Oxford: BAR International Series, 1592; 2007.

73. di Gennaro F, Pacciarelli M. Lettera di segnalazione alla Soprintendenza Archeologica del Lazio del 13.12.1976. Stud Etruschi. 1977;45: 438–439.

74. Alessandri L. Latium Vetus in the Bronze Age and Early Iron Age / Il Latium Vetus nell’età del Bronzo e nella prima età del Ferro. Oxford: BAR International Series, 2565; 2013.

75. Attema PAJ, de Haas TCA, Nijboer AJ. The Astura Project, interim report of the 2001 and 2002 campaigns of the Groningen Institute of Archaeology along the coast between Nettuno and Torre Astura (Lazio, Italy). BABesch, Bull Antieke Beschav. 2003;LXXVIII: 107–140.

76. Piccarreta F. Astura, Forma Italiae 22. 1977.

77. Attema PAJ, Alessandri L. Salt production on the Tyrrhenian coast in South Lazio (Italy) during the Late Bronze Age: its significance for understanding contemporary society,. In: Nikolov V, Bacvarov K, editors. Salz und Gold: die Rolle des Salzes im prähistorischen Europa / Salt and Gold: The Role of Salt in Prehistoric Europe. Provadia & Veliko Tarnovo; 2012. pp. 287–300.

78. Nijboer AJ, Attema PAJ, van Oortmerssen GJM. Ceramics from a Late Bronze Age saltern on the coast near Nettuno (Rome, Italy). Palaeohistoria. 2006;47/48: 141–205.

79. Alessandri L. Saracca. In: Belardelli C, Angle M, di Gennaro F, Trucco F, editors. Repertorio dei siti protostorici del Lazio - province di Roma, Viterbo e Frosinone. 2007. pp. 224–225.

80. Angle M, Guidi A. L’antica e media età del Bronzo nel Lazio. Atti della XL Riunione Scientifica dell’Istituto Italiano di Preistoria e Protostoria, Strategie di insediamento fra Lazio e Campania in età preistorica e protostorica. 2007. pp. 147–178.

81. Attema PAJ, Nijboer AJ. “Stop 4.” In: Belardelli C, Angle M, di Gennaro F, Trucco F, editors. Repertorio dei siti protostorici del Lazio - province di Roma, Viterbo e Frosinone. 2007. pp. 225–226.

82. Alessandri L. The early and Middle Bronze Age (1/2) in South and central Tyrrhenian Italy and their connections with the Avellino eruption: An overview. Quat Int. 2019;499: 161–185. doi:https://doi.org/10.1016/j.quaint.2018.08.002

83. Nijboer AJ, van der Plicht J, Bietti Sestieri AM, De Santis A. A high chronology for the early Iron Age in central Italy. Palaeohistoria. 2000;41/42: 163–176.
