# Supplementary figures and images for: Salt or fish (or salted fish)? The Bronze Age specialised sites along the Tyrrhenian coast of Central Italy: New insights from Caprolace settlement
Source: PLoS One. 2019 Nov 13;14(11):e0224435. doi: 10.1371/journal.pone.0224435 (PMC6853298; doi:10.1371/journal.pone.0224435)

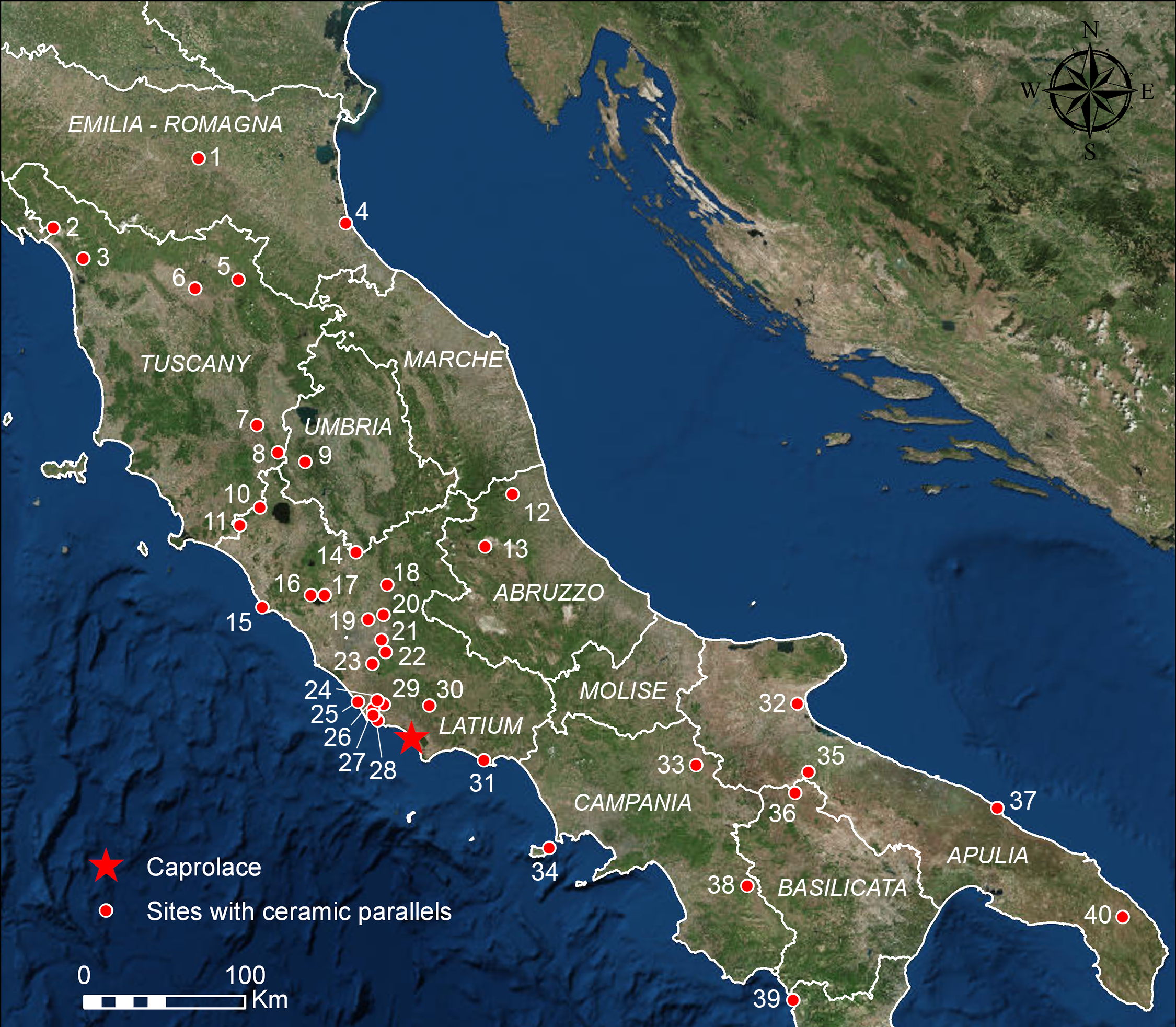

Supplement: S2 Appendix — Number of sites in S3 Appendix. Aerial photo from Esri, Digital Globe, GeoEye, Earthstar Geographics, CNES/Airbus DS, USDA, USGS, AeroGRID, IGN, and the GIS User Community. (TIF) [file pone.0224435.s002.tif]

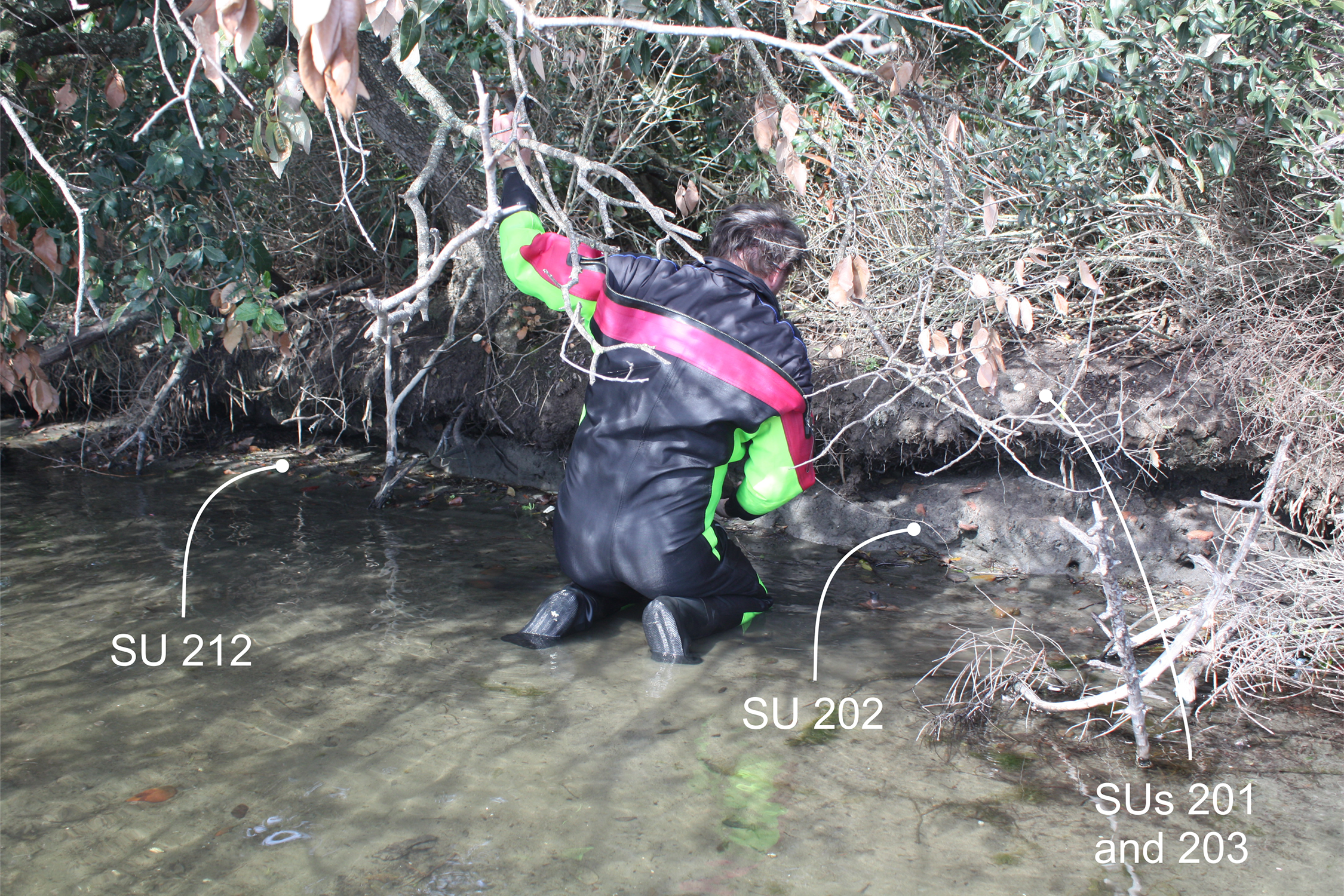

Supplement: S7 Appendix — Photo by M. F. Rolfo. (TIF) [file pone.0224435.s007.tif]

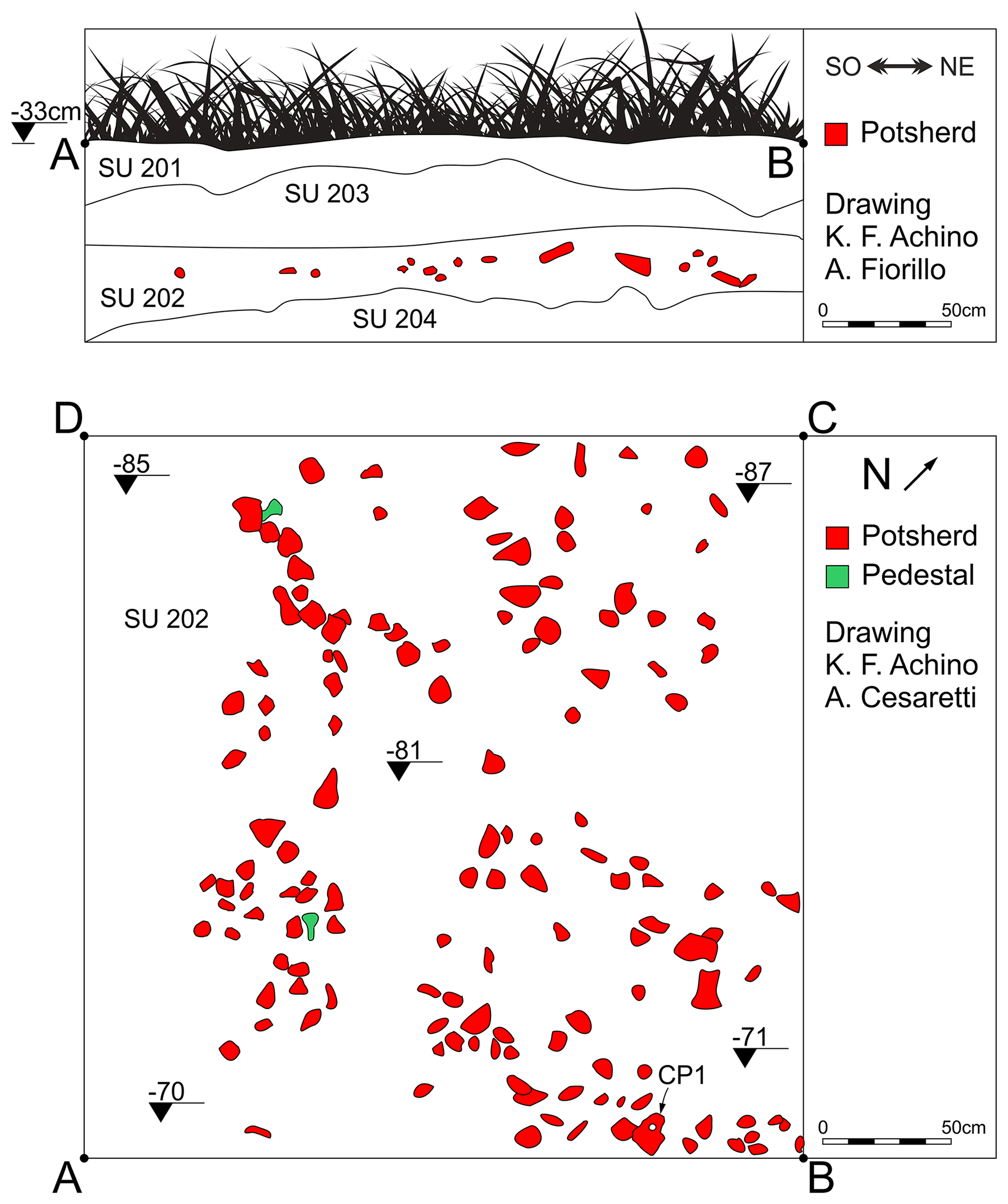

Supplement: S8 Appendix — Drawings: K. Achino, A. Fiorillo, A. Cesaretti. (TIF) [file pone.0224435.s008.tif]

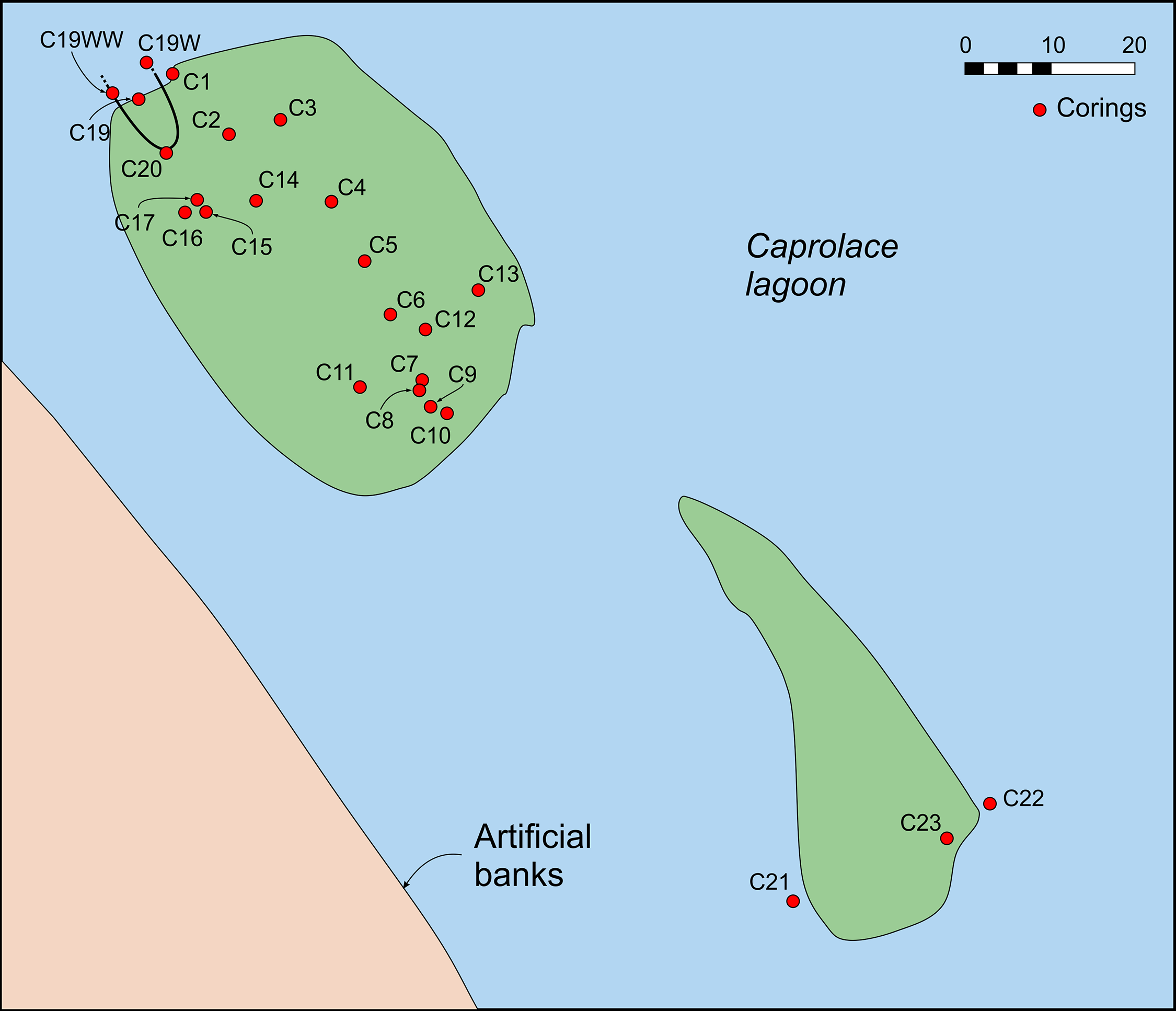

Supplement: S9 Appendix — Drawings: L. Alessandri. (TIF) [file pone.0224435.s009.tif]

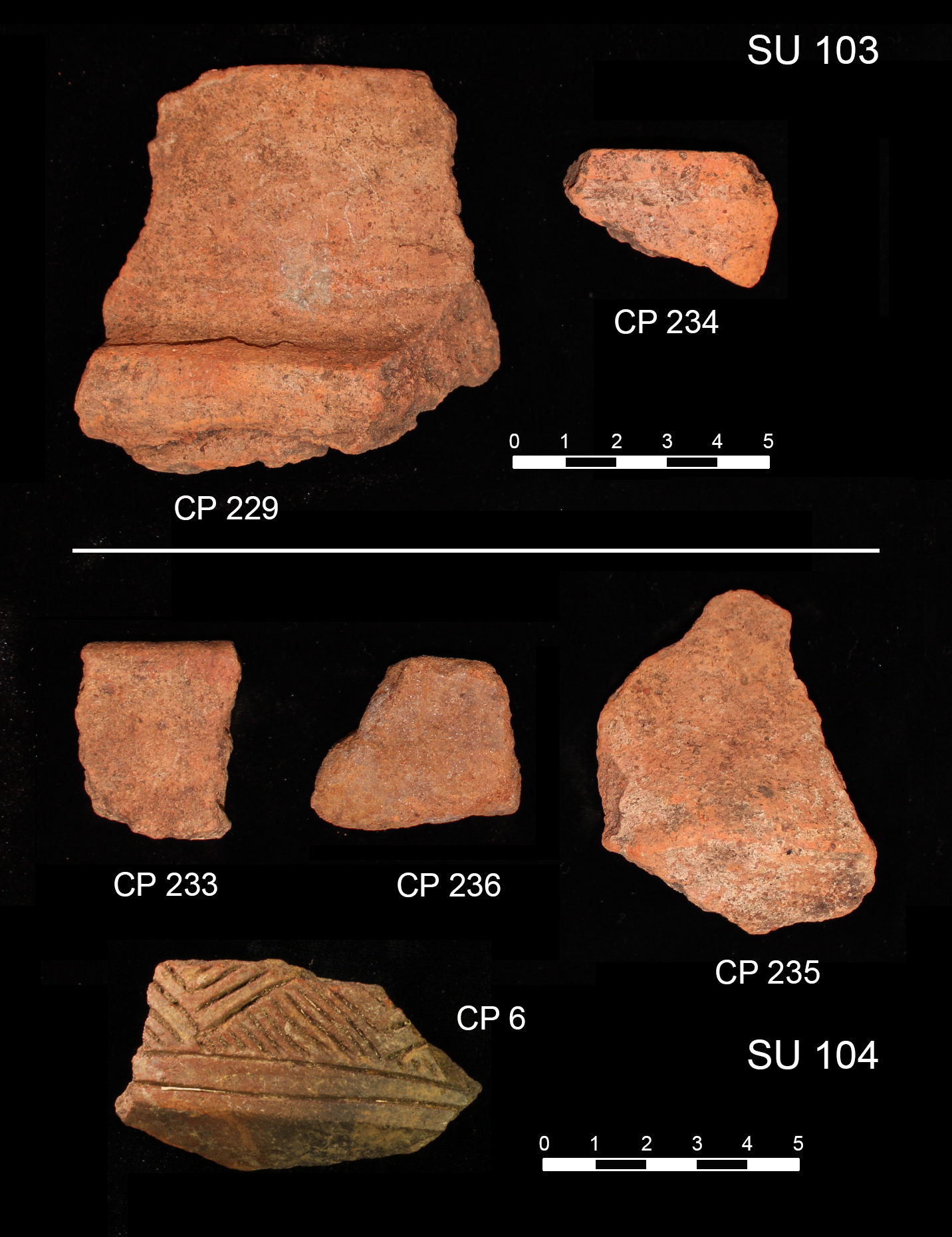

Supplement: S12 Appendix — Photo by M. F. Rolfo and A. Ferracci. (TIF) [file pone.0224435.s012.tif]
