# Supplementary material for: Salt or fish (or salted fish)? The Bronze Age specialised sites along the Tyrrhenian coast of Central Italy: New insights from Caprolace settlement
Source: PLoS One. 2019 Nov 13;14(11):e0224435. doi: 10.1371/journal.pone.0224435 (PMC6853298; doi:10.1371/journal.pone.0224435)
Supplement: S3 Appendix — (DOCX) [file pone.0224435.s003.docx]

**Caprolace potsherd parallels**

See map S2 for the location of the sites

| **N.** | **Provenance** | **Context** | **Parallel(s)** | **Parallel(s) Chronology** |  |
| --- | --- | --- | --- | --- | --- |
| CP 1 | Sound. F | SU 202 | Grotta Cardini, strato medio, [1] (Fig. 69,i) | Strato medio: MBA 2A [2] (p. 30) |  |
|  |  |  | Grotta Cardini strato superiore, [1] (Fig. 119, a, b) | Strato superiore: MBA 2B? MBA3A [2] (p. 30, Fig. 15) |  |
| CP 20 | Sound. F | SU 202 | Spallette di S. Margherita [3] (Fig.3, 3) | MBA 2 [4] (type 291A for the shape, type 512 for the grip) |  |
| CP 30 | Sound. F | SU 202 | No good parallels have been found for this miniaturistic vessel |  |  |
| CP 122 | Sound. F | SU 202 | Grotta del Beato Benincasa [5] (Fig. 24, 4) | MBA 2A [4] (type 2A) |  |
| CP 123 | Sound. F | SU 202 | Grotta del Beato Benincasa [5] (Fig. 24, 4) | MBA 2A [4] (type 2A) |  |
| CP 127 | Sound. F | SU 202 | Base of possible jar. The bases show no chronological development during the entire Bronze age and beyond |  |  |
| CP 128 | Sound. F | SU 202 | Grotta del Beato Benincasa [5] (Fig. 24, 4) | MBA 2A [4] (type 2A) |  |
|  |  |  | Fosso Foglino [6] (Fig. 129, 3) | MBA 1 [6] |  |
| CP 129 | Sound. F | SU 202 | Grotta del Beato Benincasa [5] (Fig. 24, 4) | MBA 2A [4] (type 2A) |  |
| CP 130 | Sound. F | SU 202 | Possible bar of an oven, see Monopoli, Piazza Palmieri, strato inferiore e medio [7] (Fig. 266, 298) | MBA [7] |  |
| CP 131 | Sound. F | SU 202 | Lago di Mezzano [8] (Table XXXII, M2-33) | MBA 2A [4] (type 15) |  |
| CP 132 | Sound. F | SU 202 | Base of possible jar. See CP 127 |  |  |
| Cp 133 | Sound. F | SU 202 | Grotta del Beato Benincasa [5] (Fig. 24, 4) | MBA 2A [4] (type 2A) |  |
| Cp 134 | Sound. F | SU 202 | Base of possible jar. See CP 127 |  |  |
| CP 185 | Sound. F | SU 202 | Petrosa [9] (Fig. 25, 3) | MBA 2A [4] (type 272A) |  |
| CP 186 | Sound. F | SU 202 | Spiagge Sant'Agostino [10] (Fig. 1, 5) | MBA 1-2 [11] (type 224B) |  |
|  |  |  | Villaggio delle Macine [12] (Fig. 63, 4) | MBA 1-2 [12] |  |
| CP 187 | Sound. F | SU 202 | Tecchia della Gabellaccia, with lugs [13] (Fig. 2,6) | MBA 1B-2A [4] (type 331v) |  |
|  |  |  | Anzola Emilia S. 61, SU 25 [14] (Table X, 3) | MBA 3 / RBA [14] |  |
| CP 188 | Sound. F | SU 202 | S. Maria in Belverde strati inferiori, with two holes and curved upper edge [15] (Fig. 1, 12) | MBA 1B [4] (type 494A) |  |
| CP 194 | Sound. F | SU 202 | The miniature vessel can be compared with a normal size bowl from Caprolace, surface findings [16] (Fig. 3.99, 18) | BM2A [16] |  |
| CP 199 | Sound. F | SU 202 | Valle Felici [17] (Fig. 2, 21 and 23) | MBA 1A [4] (type 67A) |  |
|  |  |  | Cavallino capanna 1a [18] (Fig. 109, 12) | MBA 1 [11] (type 425) |  |
|  |  |  | Cavallino, capanna 2 [19] (Table 25, 3) | MBA 1 [11] (type 425) |  |
| CP 200 | Sound. F | SU 202 | Spiagge Sant'Agostino [10] (Fig. 1, 5) | MBA 1-2 [11] (type 224B) |  |
| CP 201 | Sound. F | SU 202 | Grotta Cardini, strato medio [1] (Fig. 69,a) | MBA 2A [11] (type 538A) |  |
| CP 202 | Sound. F | SU 202 | Lago di Mezzano [8] (Table LXIII, M1-75406) | MBA 2A [4] (type 141) |  |
|  |  |  | Lago di Mezzano [8] (Table XXII, M1-26) | MBA 1-2 [4] (type 142) |  |
| CP 221 | Sound. F | SU 202 | see CP 194 | BM 2A |  |
| CP 222 | Sound. F | SU 202 | Grotta del Beato Benincasa, with enlarged rim and plain cord [5] (Fig. 25, 3); | MBA 1-2 [4] (type 39); |  |
|  |  |  | Spiagge S. Lorenzo [20] (n. 25) | BM 2A [21] (p. 47) |  |
| CP 223 | Sound. F | SU 202 | S. Maria di Ripalta, strato inferiore [22] (Fig. 12, 5) | MBA 2 [11] (type 425B) |  |
| CP 224 | Sound. F | SU 202 | Base of possible jar. See CP 127 |  |  |
| CP 7 | Point 64 | SU 212 | For the decoration: [23] (Table 35,8). | MBA 3B [24] (type 211D) |  |
|  |  |  | For the shape: Tane del Diavolo [23] (Table 7,2) | MBA 3 [24] (type 288) |  |
| CP 204 | Point 64 | SU 212 | Spiagge Sant'Agostino [10] (Fig. 1, 5) | MBA 1-2 [11] (type 224B) |  |
|  |  |  | Villaggio delle Macine [12] (Fig. 63, 4) | MBA 1-2 [12] |  |
| CP 207 | Point 64 | SU 212 | Foce del Marangone [25] (Fig. 1, 3) | MBA 1A [4] (type 74) |  |
| CP 208 | Point 64 | SU 212 | Vigna Grande [26] (Fig. 73, 1) | MBA 1B-2A [4] (type 13) |  |
| CP 210 | Point 64 | SU 212 | For the shape: Villaggio delle Macine [12] (Fig. 64, 7) | MBA 1B [12] |  |
|  |  |  | For the handle: Grotta Vittorio Vecchi [27] (Table XLVII, 7 and 8) | MBA 2-3 [27] |  |
| CP 211 | Point 64 | SU 212 | Base of a bowl with omphalos | MBA |  |
| CP 212 | Point 64 | SU 212 | Spiagge San Lorenzo [20] (Table I, n. 3) | BM 2A [21] (p. 47) |  |
| CP 213 | Point 64 | SU 212 | Cavallino, capanna 1a [18] (Fig. 117, 5) | MBA 1 [11] (type 475) |  |
| CP 214 | Point 64 | SU 212 | Morricone [28] (Fig. 9, 39) | MBA 1A [4] (type 115) |  |
| CP 215 | Point 64 | SU 212 | Pian Quintino, CPQ1, US 28, tg. 2 [29] (Fig. 5, 7); | MBA 3 [29] |  |
|  |  |  | Only for the handle: Grotta dello Sventatoio [30] (n. 5) | MBA 2-3 [4] (type 468) |  |
| CP 216 | Point 64 | SU 212 | Riparo Grande [31] (Fig. 2, 4) | MBA 1B [4] (type 45) |  |
| CP 217 | Point 64 | SU 212 | Spiagge Sant'Agostino [10] (Fig. 1, 5) | MBA 1-2 [11] (type 224B) |  |
|  |  |  | Villaggio delle Macine [12] (Fig. 63, 4) | MBA 1-2 [12] |  |
| CP 218 | Point 64 | SU 212 | Villaggio delle Macine [12] (Fig. 56, 3) | MBA 1-2 [12] |  |
| CP 219 | Point 64 | SU 212 | Villaggio delle Macine [12] (Fig. 56, 8) | MBA 1-2 [12] |  |
| CP 220 | Point 64 | SU 212 | Spiagge San Lorenzo [20] (Table I, n. 27) | MBA 2A [21] (p. 47) |  |
| CP 225 | Point 64 | SU 212 | Vivara, Punta Capitello, saggio B [32] (Table VI, 4.43) | MBA 3 [24] (type 266) |  |
| CP 226 | Point 64 | SU 212 | Grotta a Male, str. 4b [33] (Fig. 9, 9) | MBA 3 [24] (type 301) |  |
|  |  |  | For the knob: Le Acquarelle [26] (Fig. 74, 1) | MBA 2 [34] (type 319) |  |
| CP 227 | Point 64 | SU 212 | For the shape: Dicomano [35] (Fig. 19, 1) | MBA 1-2 [4] (type 300) |  |
|  |  |  | For the knob: Le Acquarelle [26] (Fig. 74, 1) | MBA 2 [11] (type 319) |  |
| CP 228 | Point 64 | SU 212 | Grotta a Male, str. 2b (Pannuti 1969, Fig. 47, 4) | MBA 1-2 [34] (type 360) |  |
|  |  |  | La Campana [36] (Fig. 131.1, 8 and 11) | MBA 1-2 (3?) [36] |  |
| CP 238 | Point 64 | SU 212 | Spiagge San Lorenzo [20] (Table I, n. 27) | MBA 2A [21] (p. 47) |  |
| CP 61 | Sound. G | SU 103 | Spiagge San Lorenzo [20] (Table I, n. 22) | MBA 2A [21] (p. 47) |  |
| CP 206 | Sound. G | SU 103 | Valdroni [36] (Fig. 27.1, 11) | The context is dated MBA 2A and FBA [36] |  |
| CP 209 | Sound. G | SU 103 | Half-conical vessels are widespread during all Bronze Age and beyond. For MBA 3 similar specimens see for example: Castiglione [37] (Fig. 81, "scodella troncoconica") | MBA 3 [37] |  |
| CP 229 | Sound. G | SU 103 | Moje [28] (Fig. 4, 25) | MBA 1-3 [28] |  |
| CP 230 | Sound. G | SU 103 | Base of possible jar. See CP 127 |  |  |
| CP 234 | Sound. G | SU 103 | Moje [28] (Fig. 4, 25) | MBA 1-3 [28] |  |
| CP 239 | Sound. G | SU 103 | See CP 209 | MBA 3 |  |
| CP 240 | Sound. G | SU 103 | Base of possible jar. See CP 127 |  |  |
| CP 241 | Sound. G | SU 103 | See CP 209 | MBA 3 |  |
| CP 242 | Sound. G | SU 103 | Lavello, Ipogeo 1036 [38] (Fig. 6) | MBA 1-2 [38] |  |
| CP 243 | Sound. G | SU 103 | Base of possible jar. See CP 127 |  |  |
| CP 245 | Sound. G | SU 103 | Base of possible jar. See CP 127 |  |  |
| CP 248 | Sound. G | SU 103 | Base of possible jar. See CP 127 |  |  |
| CP 252 | Sound. G | SU 103 | Coppa Nevigata, coll. A. Boschi [39] (n. 10, decorated) | MBA 3B [24] (type 143) |  |
| CP 6 | Sound. G | SU 104 | For the shape: La Starza [23] (Table 36,4) | MBA 3 [24] (type 112) |  |
|  |  |  | For the shape: Coccioli, sett. F, 5-6, tgl. I-IV [23] (Table 30,3) | MBA 3 [24] (type 112) |  |
|  |  |  | For the shape: Grotta dello Sventatoio [30] (n. 7) | MBA 3 [24] (type 112) |  |
| CP 190 | Sound. G | SU 104 | Valdroni [36] (Fig. 27.1, 11) | The context is dated MBA 2A and FBA [36] |  |
| CP 191 | Sound. G | SU 104 | La Campana [36] (Fig. 131.2, 16) | The context is dated MBA 2, MBA 3? [36] |  |
| CP 192 | Sound. G | SU 104 | Torre del Padiglione, nord [40] (Fig. 4, 1) | MBA 3 [40] |  |
|  |  |  | San Giacomo [41] (Fig. 136, 5) | MBA 1-2 [41] |  |
| CP 195 | Sound. G | SU 104 | Grotta di Carli [42] (Fig. 3, 7) | The context is dated between Neolithic and MBA 3 [42] |  |
| CP 196 | Sound. G | SU 104 | Villaggio delle Macine [12] (Fig. 64, 2) | MBA 1-2 [12] |  |
| CP 231 | Sound. G | SU 104 | Spiagge San Lorenzo [20] (Table I, n. 26) | MBA 2A [21] (p. 47) |  |
| CP 232 | Sound. G | SU 104 | See CP 209 | MBA 3 |  |
| CP 233 | Sound. G | SU 104 | See CP 209 | MBA 3 |  |
| CP 235 | Sound. G | SU 104 | Base of possible jar. See CP 127 |  |  |
| CP 236 | Sound. G | SU 104 | See CP 209 | MBA 3 |  |
| CP 237 | Sound. G | SU 104 | Base of possible jar. See CP 127 |  |  |
| CP 244 | Sound. G | SU 104 | Base of possible jar. See CP 127 |  |  |
| CP 246 | Sound. G | SU 104 | Briquetage bar? |  |  |
| CP 247 | Sound. G | SU 104 | Base of possible jar. See CP 127 |  |  |
| CP 249 | Sound. G | SU 104 | Base of possible jar. See CP 127 |  |  |
| CP 250 | Sound. G | SU 104 | See CP 195 | Neolithic to MBA 3 (for the context) |  |
| CP 251 | Sound. G | SU 104 | Base of possible jar. See CP 127 |  |  |

**References**

1. Bernabò Brea L, Biddittu I, Cassoli PF, Cavalier M, Scali S, Tagliacozzo A, et al. La Grotta Cardini (Praia a Mare - Cosenza): giacimento del Bronzo. Roma; 1989.

2. Pacciarelli M. Dal villaggio alla città. La svolta protourbana del 1000 a.C. nell’Italia tirrenica. Firenze; 2001.

3. Cardarelli A. Siti di passaggio alla media età del Bronzo nel Lazio. Archeol Laz. 1979;II: 139–147.

4. Cocchi Genick D. Classificazione tipologica e processi storici: le ceramiche della facies di Grotta Nuova. Viareggio; 2001.

5. Radi G. La Grotta del Beato Benincasa nel quadro delle culture dal Neolitico all’età del Bronzo in Toscana. Pisa; 1981.

6. Alessandri L. Fosso Foglino. In: Belardelli C, Angle M, di Gennaro F, Trucco F, editors. Repertorio dei siti protostorici del Lazio - province di Roma, Viterbo e Frosinone. 2007. pp. 218–219.

7. Cinquepalmi A. Monopoli, centro storico. In: Cinquepalmi A, Radina F, editors. Documenti dell’età del Bronzo Ricerche lungo il versante adriatico pugliese. Fasano; 1998. pp. 109–124.

8. Franco MC. L’insediamento preistorico del Lago di Mezzano. Roma; 1982.

9. Morandi R, Sarti L. Industria fittile. In: Sarti L, editor. Petrosa Un insediamento dell’età del Bronzo a Sesto Fiorentino. Montelupo Fiorentino; 1994. pp. 46–75.

10. Guidi A. Rinvenimenti preistorici nel territorio della Soprintendenza del Lazio. Archeol Laz. 1980;10: 38–42.

11. Damiani I. La facies protoappenninica. In: Cocchi Genick D, editor. Aspetti culturali della media età del Bronzo in Italia centro-meridionale. 1995. pp. 398–428.

12. Angle M, Lugli F, Molinaro A, Rosa C, Zarattini A, Cattani L, et al. Villaggio delle Macine. In: Belardelli C, Angle M, di Gennaro F, Trucco F, editors. Repertorio dei siti protostorici del Lazio - province di Roma, Viterbo e Frosinone. 2007. pp. 173–181.

13. Radi G. La Tecchia della Gabellaccia (Carrara). Note paletnologiche. Atti della Soc Toscana di Sci Nat Mem. 1976;LXXXIII: 81–102.

14. Argentina F, Desantis P, Finotelli F, Schneider F. Il sito dell’età del Bronzo di Anzola dell’Emilia. Quad della Rocca. 2002;9: 39–80.

15. Cuda MT, Sarti L. Nuove ricerche a Belverde di Cetona (Siena). Rass di Archeol. 1992;10: 385–392.

16. Alessandri L. L’occupazione costiera protostorica del Lazio centromeridionale. Oxford: BAR International Series, 1592; 2007.

17. Bermond Montanari G. L’insediamento di Valle Felici presso Cervia e la media età del Bronzo in Romagna. Rass di Archeol. 1992;10: 175–383.

18. Pancrazzi O. Cavallino I. Galatina; 1979.

19. Ingravallo E. L’insediamento protoappenninico di Cavallino. Stud di Antich. 1990;6: 59–100.

20. Morandini A. Gli insediamenti costieri in età protostorica nel Lazio meridionale. Latium. 1999;XVI: 5–47.

21. Cocchi Genick D. Grotta Nuova : la prima unità culturale attorno all’Etruria protostorica. Viareggio Lucca [Italy]: M. Baroni; 2002.

22. Nava ML. S. Maria di Ripalta (Cerignola): prima campagna di scavi. Atti del 2° Convegno sulla Preistoria - Protostoria - Storia della Daunia. San Severo; 1982. pp. 185–191.

23. Macchiarola I. La ceramica appenninica decorata. Roma; 1987.

24. Macchiarola I. La facies appenninica. In: Cocchi Genick D, editor. Aspetti culturali della media età del Bronzo in Italia centro-meridionale. 1995. pp. 441–463.

25. Fugazzola Delpino MA. Testimonianze di cultura appenninica nel Lazio. Firenze: Sansoni; 1976.

26. Fugazzola Delpino MA, Lombardi AL. Vigna Grande, Vigna di Valle. Palafitte mito e realtà. Verona; 1983. pp. 230–232, 233–234.

27. Guidi A, Rosini L. Materiali protostorici dalla grotta Vittorio Vecchi (Sezze Romano, LT). Oxford: BAR International Series, 2919; 2019.

28. Filippi G, Pacciarelli M. Materiali protostorici dalla Sabina tiberina. Magliano Sabina; 1991.

29. Angle M, Micarelli F, Spera A. Recenti rinvenimenti dell’età del Bronzo in loc. Pian Quintino (Colonna, Roma). 2009. pp. 203–212.

30. Angle M, Gianni A, Guidi A. La Grotta dello Sventatoio (S. Angelo Romano, Roma). Rass di Archeol. 1992;10: 720–721.

31. Cocchi Genick D. La media età del Bronzo al Riparo Grande (Camaiore, Lucca). Origini. 1992;XV: 283–300.

32. Damiani I, Marazzi M, Pacciarelli M, Saltini A. L’insediamento preistorico di Vivara. Napoli antica. 1985. pp. 35–43.

33. Pannuti S. Gli scavi di Grotta a Male presso l’Acquila. Bull di Paletnologia Ital. 1969;78: 147–247.

34. Cocchi Genick D. La facies di Grotta Nuova. In: Cocchi Genick D, editor. Aspetti culturali della media età del Bronzo in Italia centro-meriidonale. 1995. pp. 364–397.

35. Sarti L. L’insediamento dell’età del Bronzo di Dicomano (Firenze). Riv di Sci Preist. 1980;XXXV, 1–2: 183–247.

36. Alessandri L. Latium Vetus in the Bronze Age and Early Iron Age / Il Latium Vetus nell’età del Bronzo e nella prima età del Ferro. Oxford: BAR International Series, 2565; 2013.

37. Bietti Sestieri AM, De Santis A. Protostoria dei popoli latini. Milano; 2000.

38. Nava ML. Elementi cultuali dell’Età del Bronzo: il caso dell’ipogeo 1036 di Lavello. In: Bonaudo R, Cerchiai L, Pellegrino C, editors. Tra Etruria, Lazio e Magna Grecia: indagini sulle necropoli. Paestum; 2009. pp. 11–20.

39. Belardelli C. Coppa Nevigata (Foggia): materiali dagli scavi Quagliati e dalla collezione Achille Boschi. Rass di Archeol. 1992;10: 736–737.

40. Angle M. Torre del Padiglione. In: Belardelli C, Pascucci P, editors. Repertorio dei siti protostorici del Lazio - province di Rieti e Latina. Roma; 1996. pp. 59–61.

41. Angle M. San Giacomo. In: Belardelli C, Angle M, di Gennaro F, Trucco F, editors. Repertorio dei siti protostorici del Lazio - province di Roma, Viterbo e Frosinone. 2007. pp. 221–224.

42. Casi C, Mieli G. Nuovi dati sulla Grotta di Carli di Ischia di Castro (VT). In: Negroni Catacchio N, editor. Atti del terzo incontro di studi Preistoria e Protostoria in Etruria Protovillanoviani e/o protoetruschi. Firenze: OCTAVO F. Cantini editore; 1998. pp. 411–419.
